# Supplementary material for: Penaeid shrimp genome provides insights into benthic adaptation and frequent molting
Source: Nat Commun. 2019 Jan 21;10:356. doi: 10.1038/s41467-018-08197-4 (PMC6341167; doi:10.1038/s41467-018-08197-4)
Supplement: Supplementary file 1 — Supplementary Information [file 41467_2018_8197_MOESM1_ESM.pdf]

Decoding the penaeid shrimp genome provides insights into benthic adaptation, frequent molting and breeding impacts

Zhang et al.

Supplementary Information

Supplementary Figures

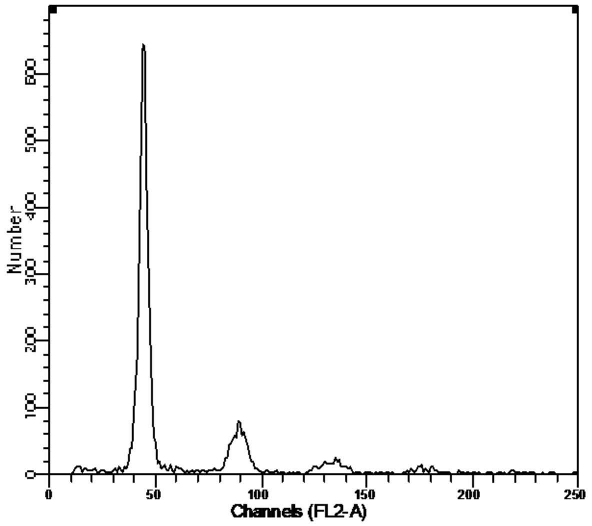

Supplementary Figure 1. Flow cytometry results for measuring the genome size of *L. vannamei*.

Based on previous reports[1], the genome size of *L. vannamei* was estimated to be approximately 71.5±5.2% of the human genome (C value = 3.5pg). We validated the result by flow cytometry, with the *L. vannamei* genome size identified as 2.45 Gb.

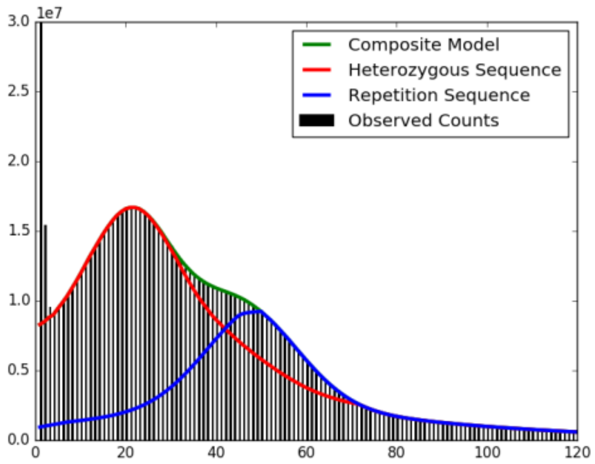

Supplementary Figure 2. K-mer distribution of the *L .vannamei* genome sequences.

"Composite Model" indicates the composite K-mer distribution of the genome of *L. vannamei*, "Heterozygous Sequence" indicates the K-mer distribution of simulated heterozygous sequences, "Repetition Sequence" indicates the K-mer distribution of simulated repetitive sequences. K-mer analysis estimated the genome size of *L .vannamei* to be 2.6 Gb.

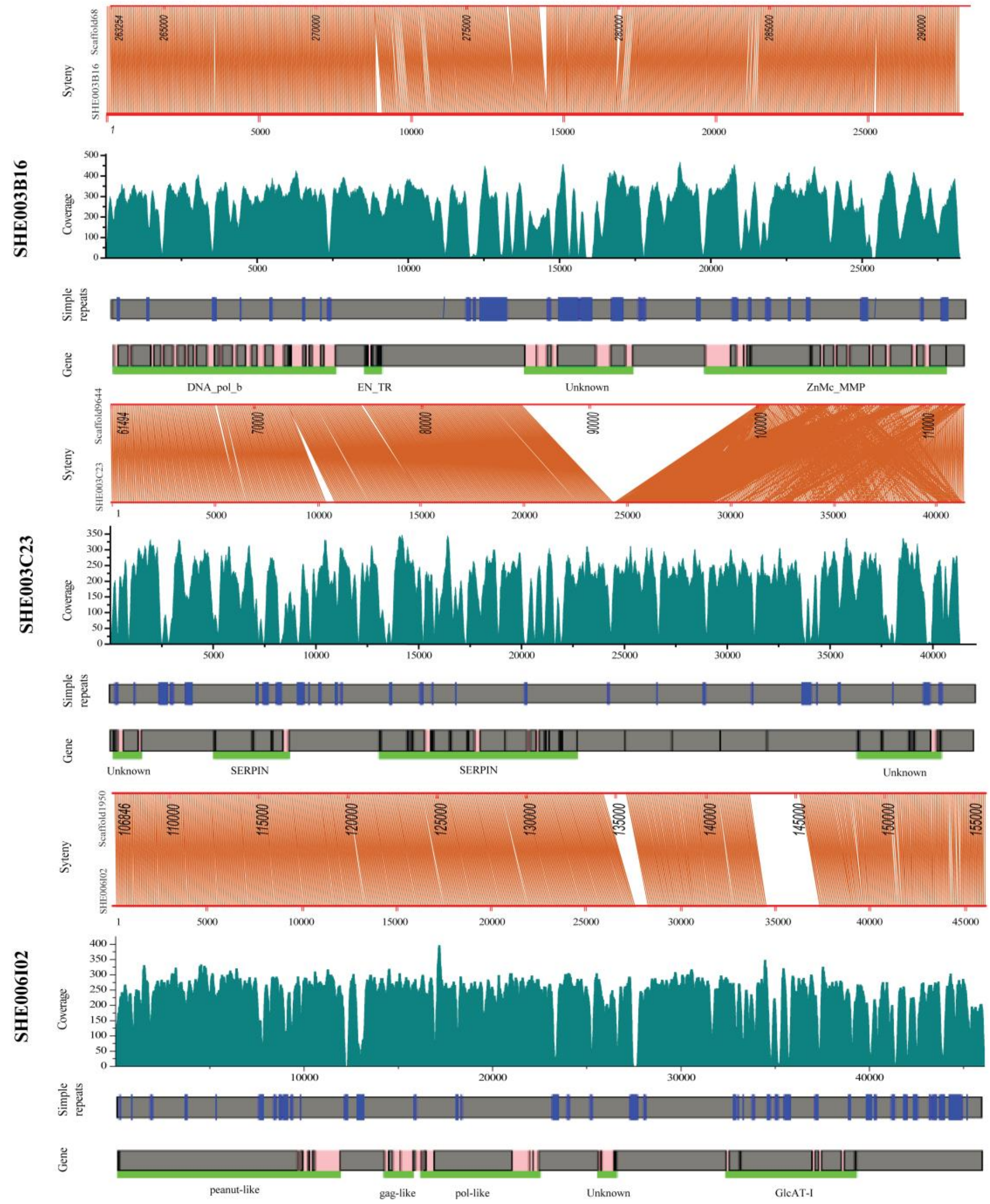

Supplementary Figure 3. The assembly accuracy of the *L. vannamei* genome assessed by three full BAC sequences.

"Synteny" indicates the synteny between the assembly scaffolds and BAC clones sequenced by the Sanger method, "Coverage" indicates the Illumina coverage along each assembly scaffold, "Simple repeats" indicates the distribution of SSRs along each assembly scaffold, "Gene" indicates the distribution of gene features along each assembly scaffold, pink block indicates exons, and green block indicates genes. Low sequencing coverage of the Illumina data were detected at the SSR regions, making the assembly highly fragmental. BAC clones were completely covered by corresponding single scaffold with high synteny, suggesting the high accuracy of the assembly.

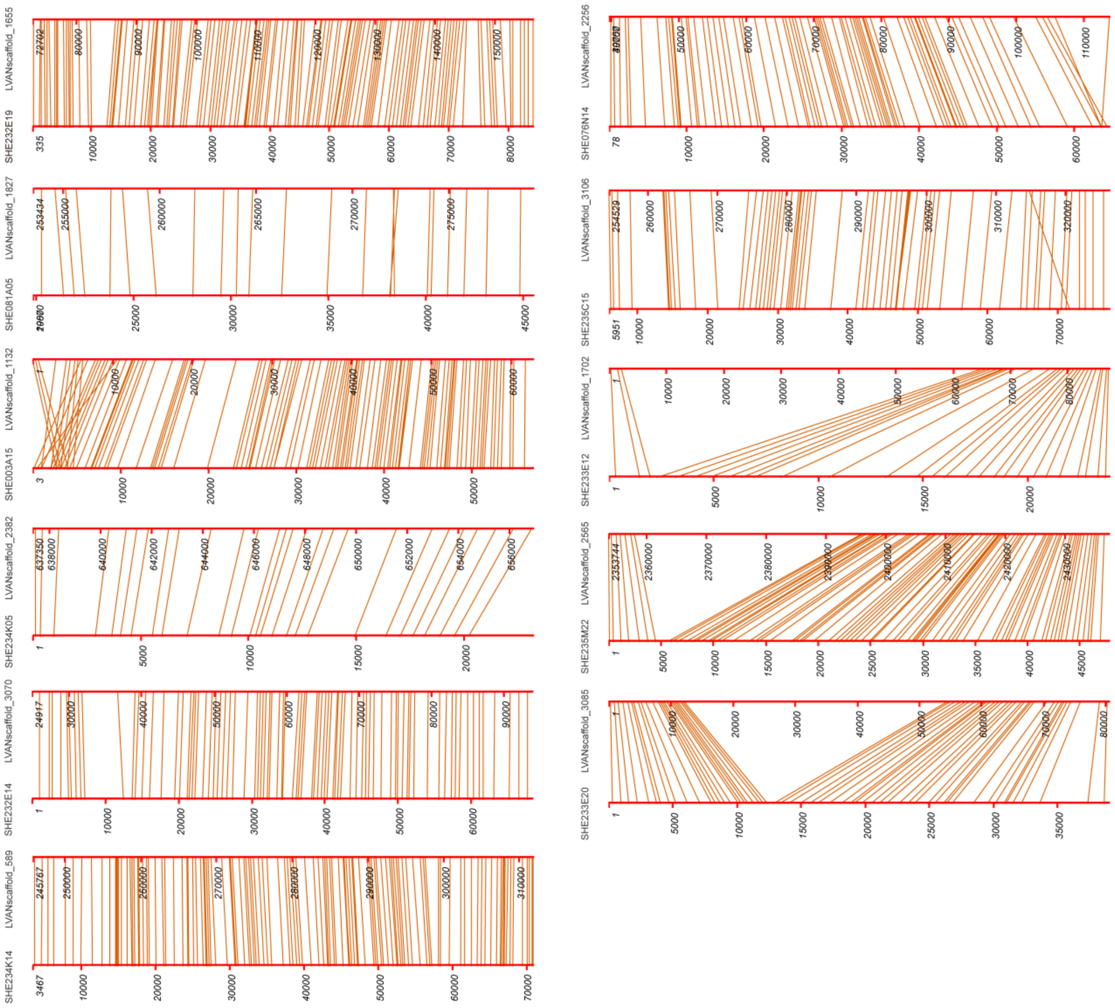

Supplementary Figure 4. The assembly accuracy of the *L. vannamei* genome assessed by 11 PacBio sequenced BAC sequences.

BAC clones were completely covered by corresponding single scaffold with high synteny, suggesting the high accuracy of the assembly.

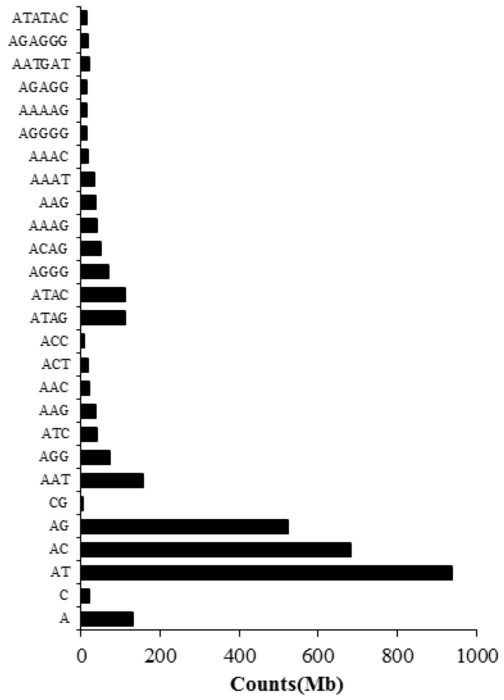

Supplementary Figure 5. Distribution of different types of SSRs in the *L. vannamei* genome.

Dinucleotide repeats are the most prevalent type of SSRs in *L. vannamei* genome, the $(AT)_n$, $(AC)_n$, and $(AG)_n$ account for 81.40% of total SSRs.

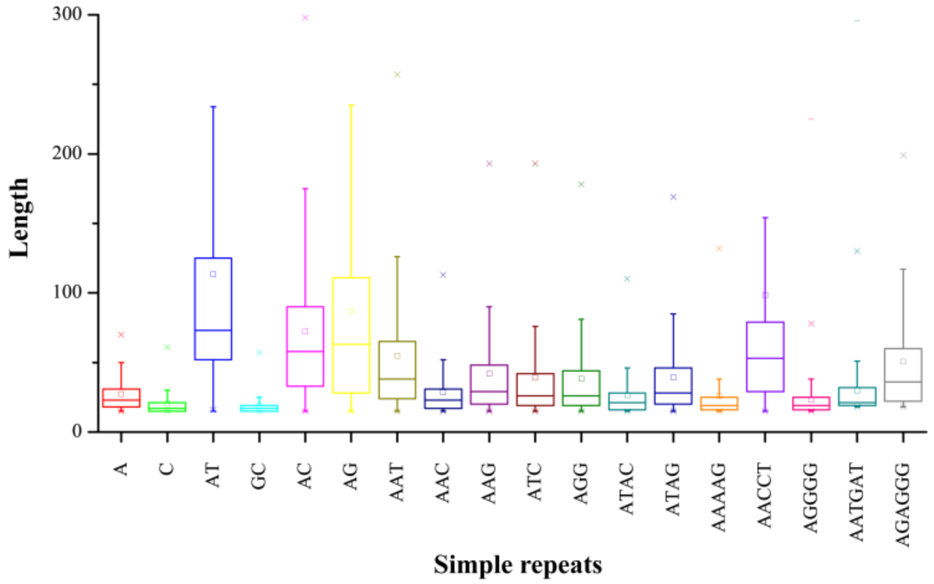

Supplementary Figure 6. Box plot of the length of SSRs.

The average length of dinucleotide repeats, including $(AT)_n$, $(AC)_n$, and $(AG)_n$, are longer than other SSRs, followed by a pentanucleotide repeat $(AACCT)_n$, with an average length of 97 bp, even though it only accounts for 0.07% of the genome.

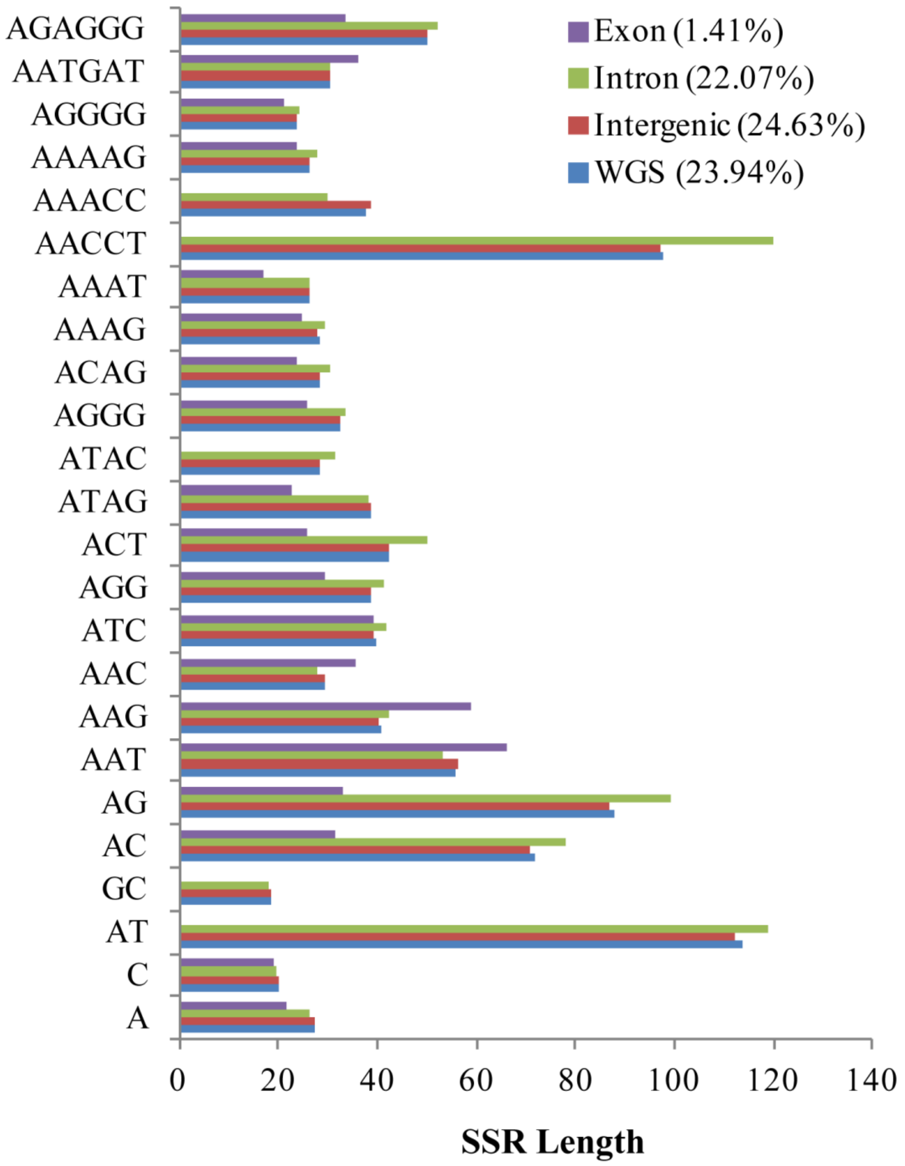

Supplementary Figure 7. The distribution of SSRs in the genome of *L. vannamei*. The distribution of SSRs was similar in the exon, intron, and intergenic regions. The length of $(AACCT)_n$ in the introns are relatively longer than those in other genomic regions.

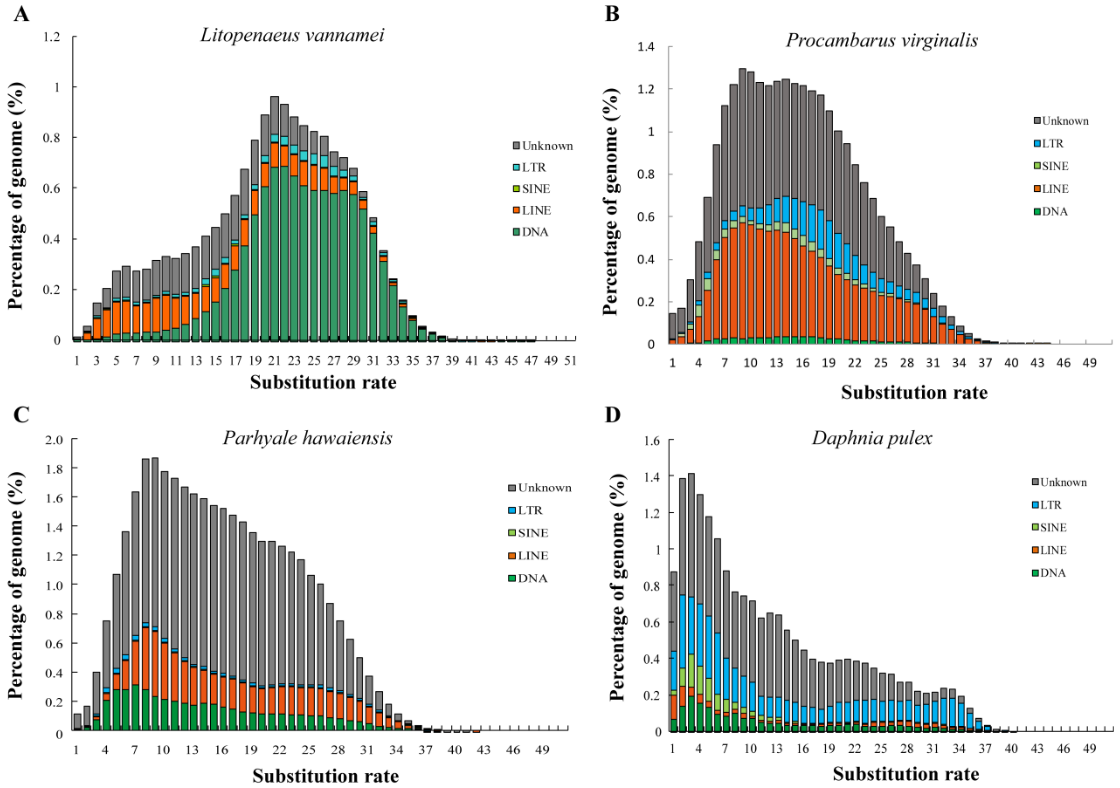

Supplementary Figure 8. Age distribution of repeats in the crustacean genomes.

The substitution rates were calculated between the genomic and repeat consensus sequences. The two transposable element expansion peaks were found in four genomes, (A) *L. vannamei*, (B) *P. virginalis*, (C) *P. hawaiensis*, and (D) *D. pulex*. In comparison to the other three crustaceans, *L. vannamei* has a major expansion of transposable elements at older age (substitution rate of 19-33).

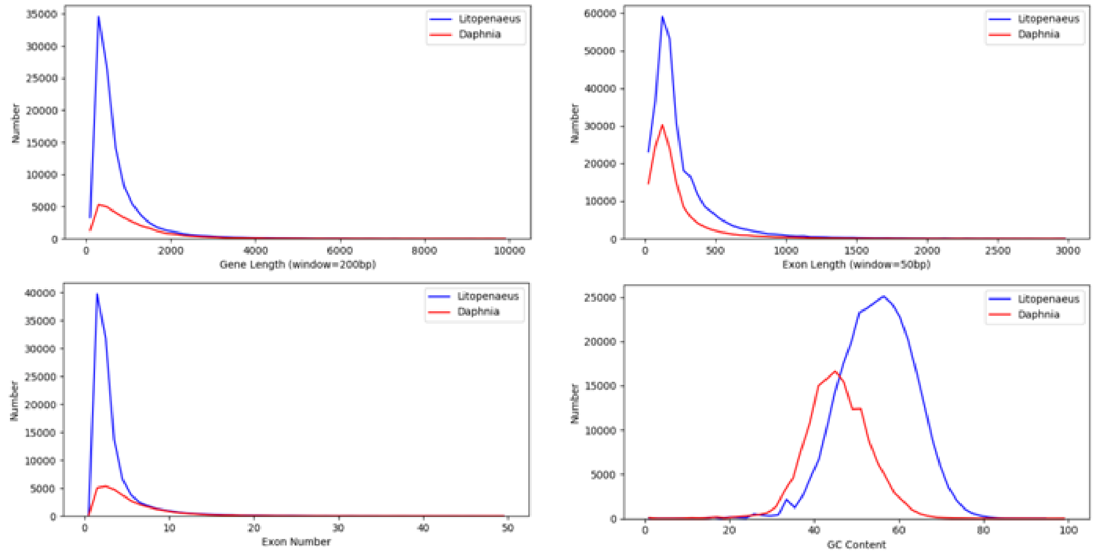

Supplementary Figure 9. Gene structure comparison between *L. vannamei* and *D. pulex*.

Four gene features, including gene length, exon length, exon number, and GC content of genes, were compared between *L. vannamei* and *D. pulex*. *L. vannamei* showed

similar distribution of gene length, exon length, and exon number with *D. pulex*, but have higher GC content than *D. pulex*.

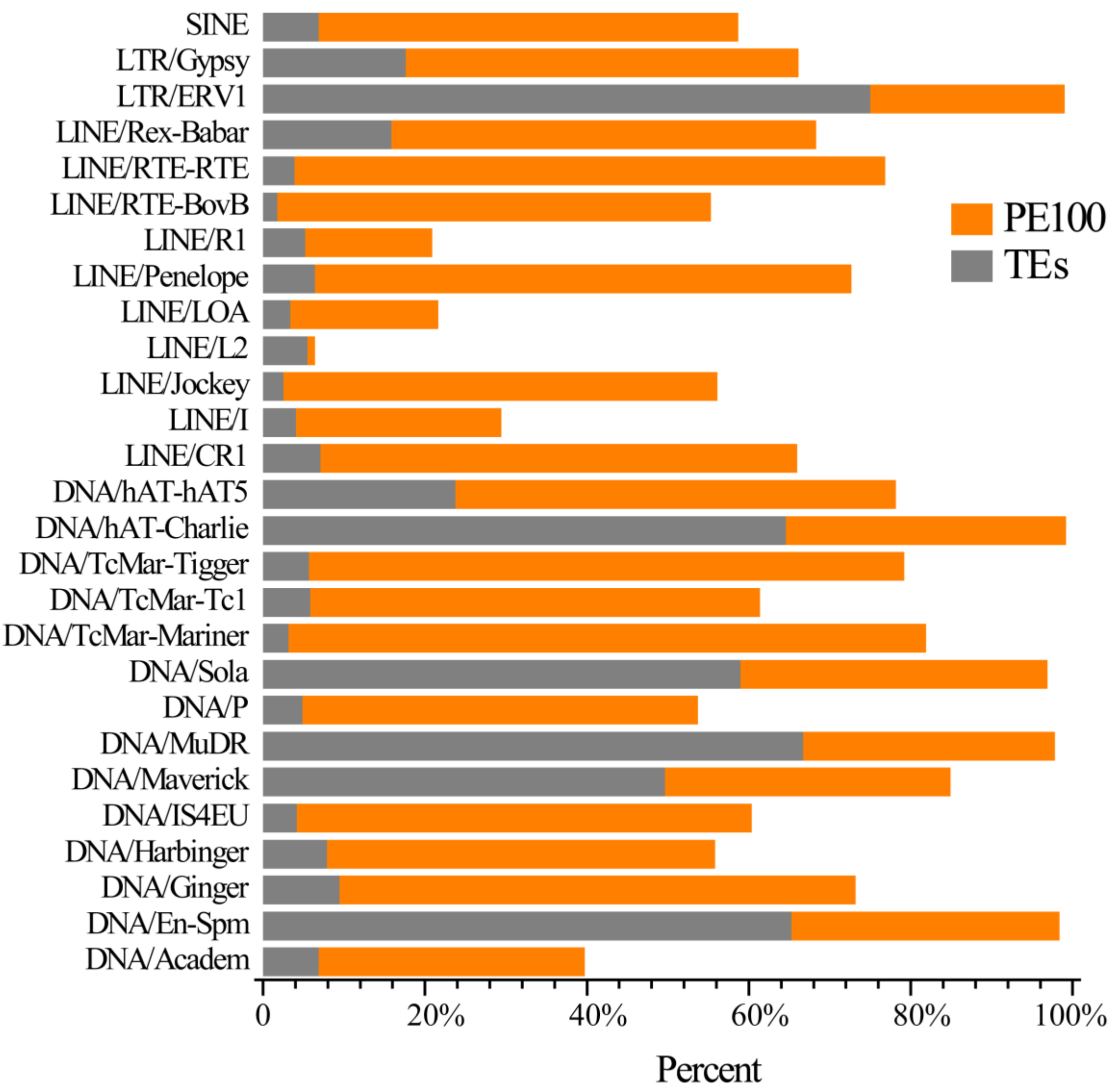

Supplementary Figure 10. The distribution of SSRs on TEs.

Most of TEs and their up- or down-stream sequences contain SSRs. "PE100" indicates the up- or down-stream sequences contain SSRs, and "TEs" indicates TEs itself contain SSRs.

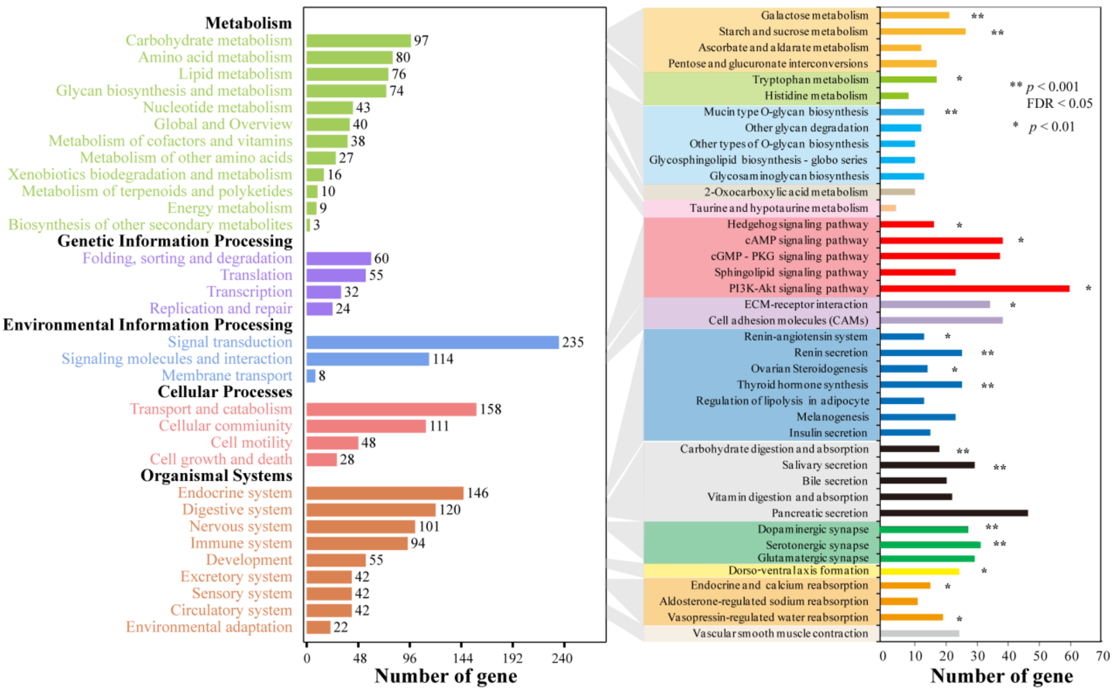

Supplementary Figure 11 KEGG enrichment of the species-specific genes of *L. vannamei*.

The species-specific genes of *L. vannamei* were particularly enriched in the KEGG terms of carbohydrate metabolism, signal transduction, endocrine system, digestive system, nervous system, and immune system.

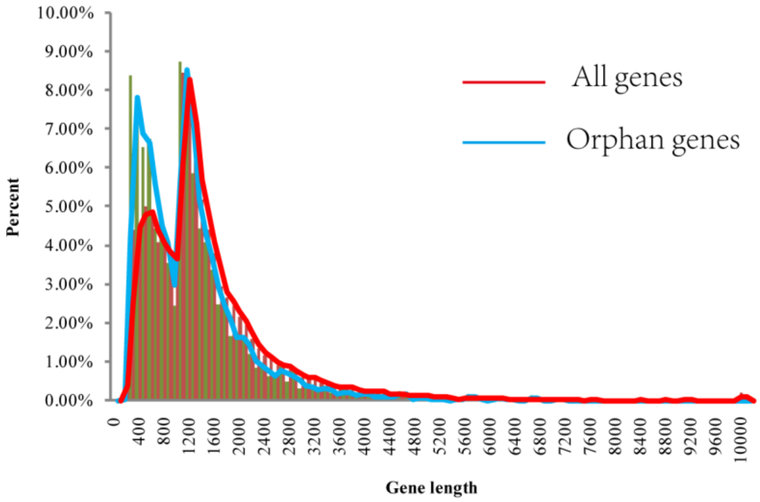

Supplementary Figure 12. Distribution of orphan genes length in the *L. vannamei* genome.

A group of orphan genes (1,282) with the gene length significantly shorter than the average length of all genes.

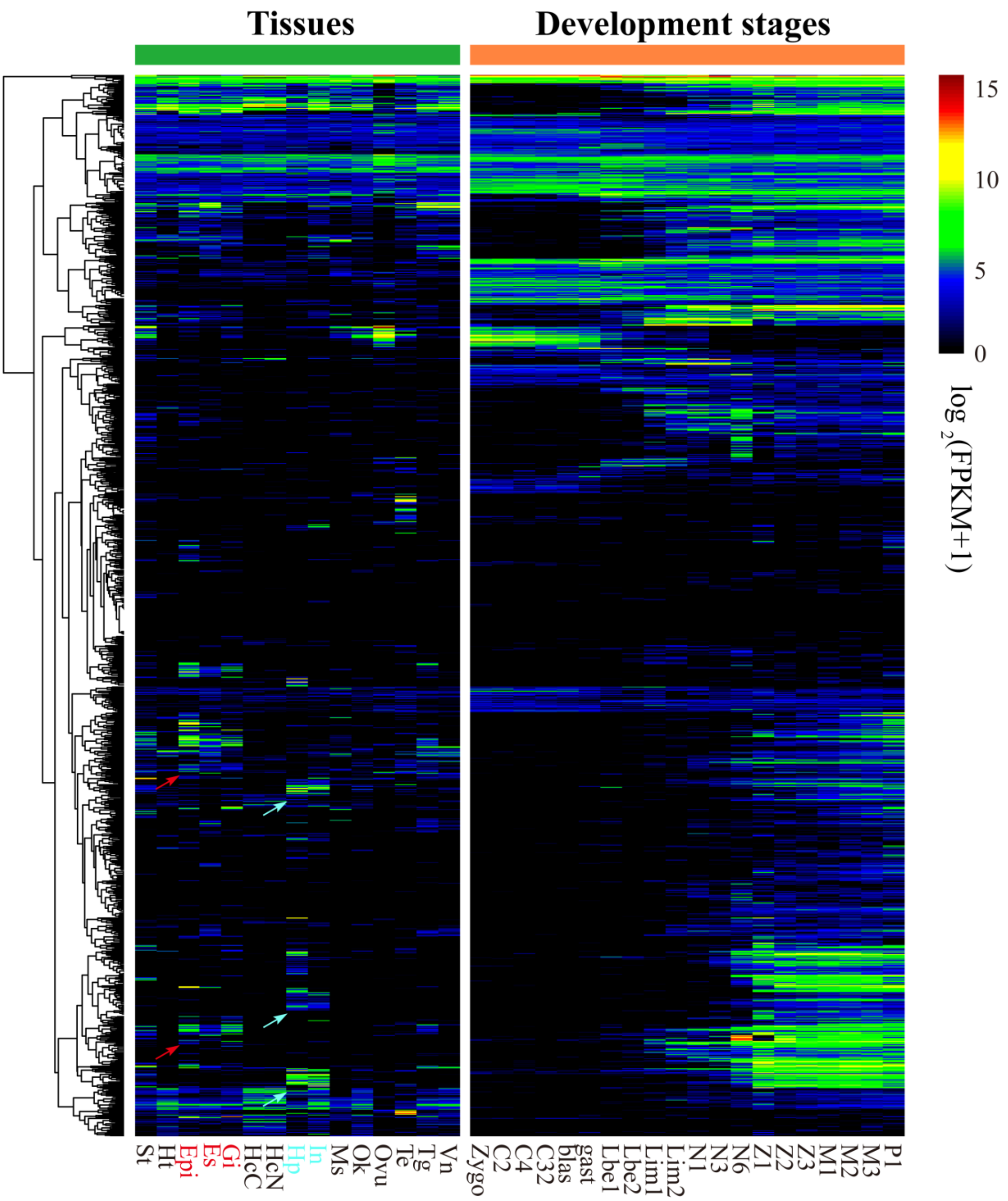

Supplementary Figure 13. The expression patterns of orphan genes in different tissues and developmental stages.

These orphan genes displayed special temporal and spatial expression patterns that most of them were up-regulated at the stage of limb bud embryo, and some of them specifically expressed in intestinal, hepatopancreas, eyestalk, cuticle and gill, implying that these orphan genes play an important role during larval development. Tissues: Hc hemocyte, Ms muscle, In intestine, Ov ovary, St stomach, Oka lymphoid organ, Gi gill, Hp hepatopancreas, Te testis, Es eyestalk, Epi epidermis, Tg thoracic ganglion, Vn ventral ganglion, Ht heart.

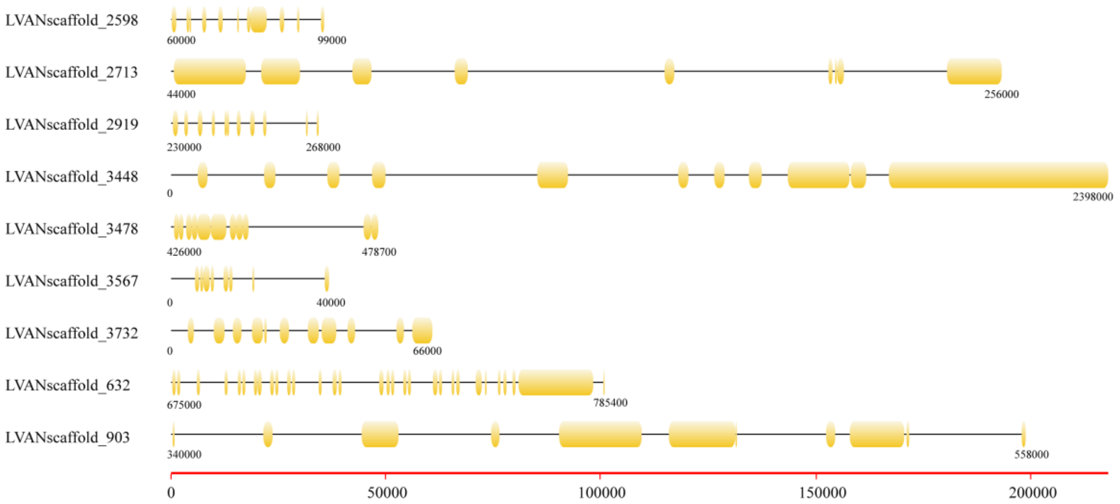

Supplementary Figure 14. The tandemly duplicated genes in the *L. vannamei* genome.

Every yellow box represents a gene, and the genes on the same scaffold are tandemly duplicated.

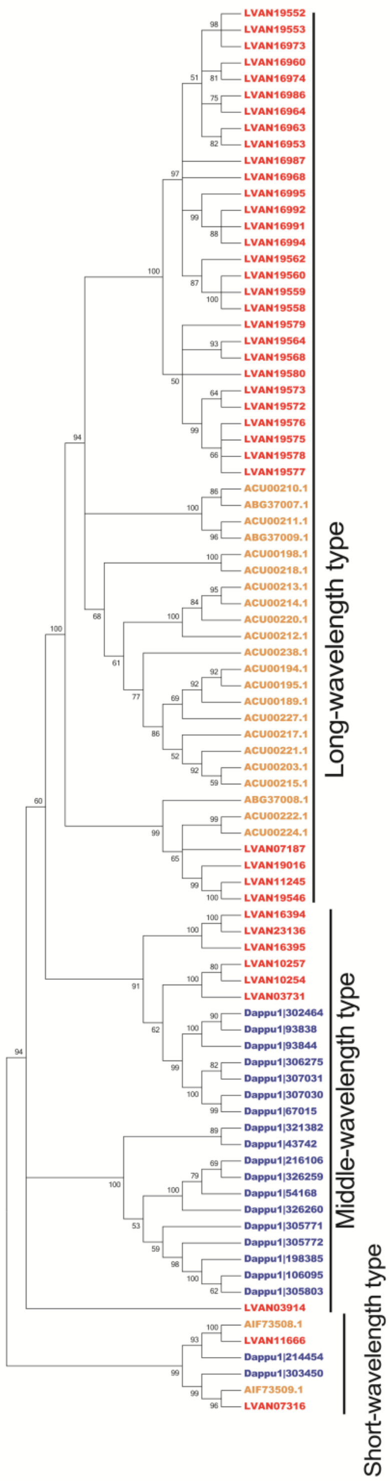

Supplementary Figure 15. Phylogenetic relationship of visual-type opsin genes in three species of crustacean, including *L. vannamei* (red text) and mantis shrimps (orange text) with benthic living habitat, and *Daphnia pulex* (blue text) with planktonic living habitat.

The neighbor-joining phylogenetic tree is shown. A bootstrap analysis of 1,000 replications was carried out. On each node, bootstrap values are indicated. Accession numbers or gene IDs are labeled. Classification of the opsin genes is indicated at the right side.

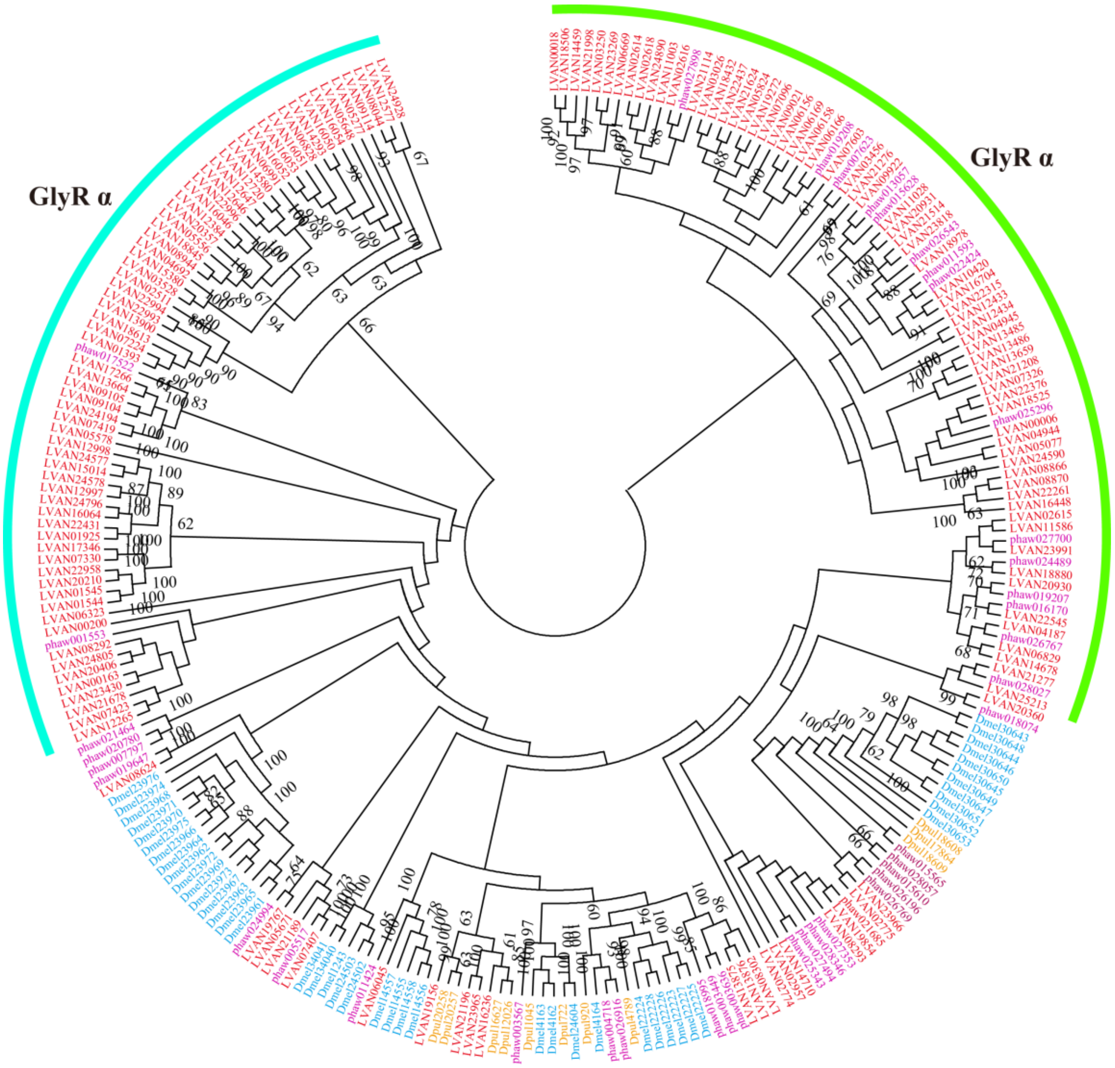

Supplementary Figure 16. Expansion of the glycine receptor gene family in the *L. vannamei* genome.

Totally 148 GlyR alpha subunit genes were found expanded in the *L. vannamei* genome. The two GlyR alpha groups are shown in blue and green.

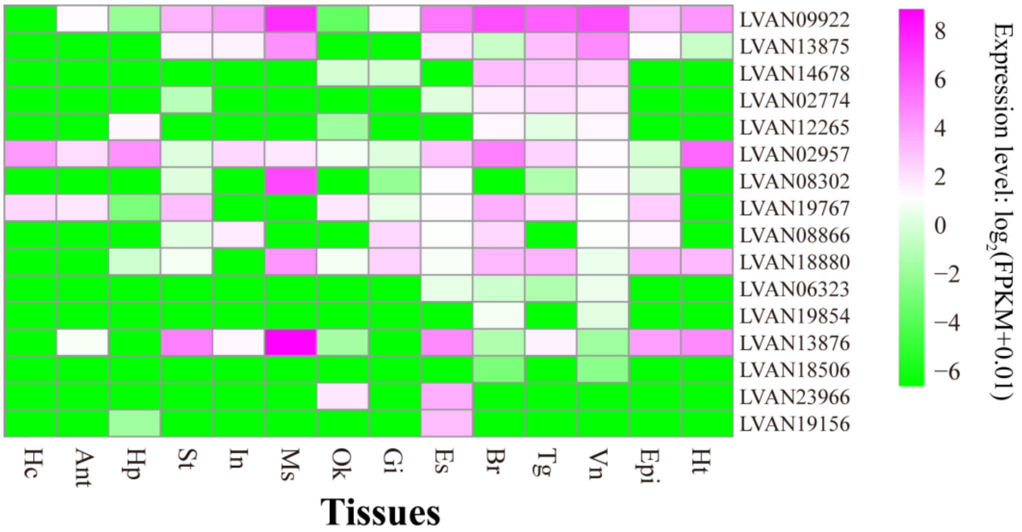

Supplementary Figure 17. The expressions of glycine receptor genes in different tissues.

GlyRs were mainly expressed in nervous system as well as in muscle and intestinal tissues. Hc, hemocyte. Ms, muscle. In, intestine. St, stomach. Oka, lymphoid organ. Gi, gill. Hp, hepatopancreas. Es, eyestalk. Epi, epidermis. Tg, thoracic ganglion. Vn, ventral ganglion. Ht, heart.

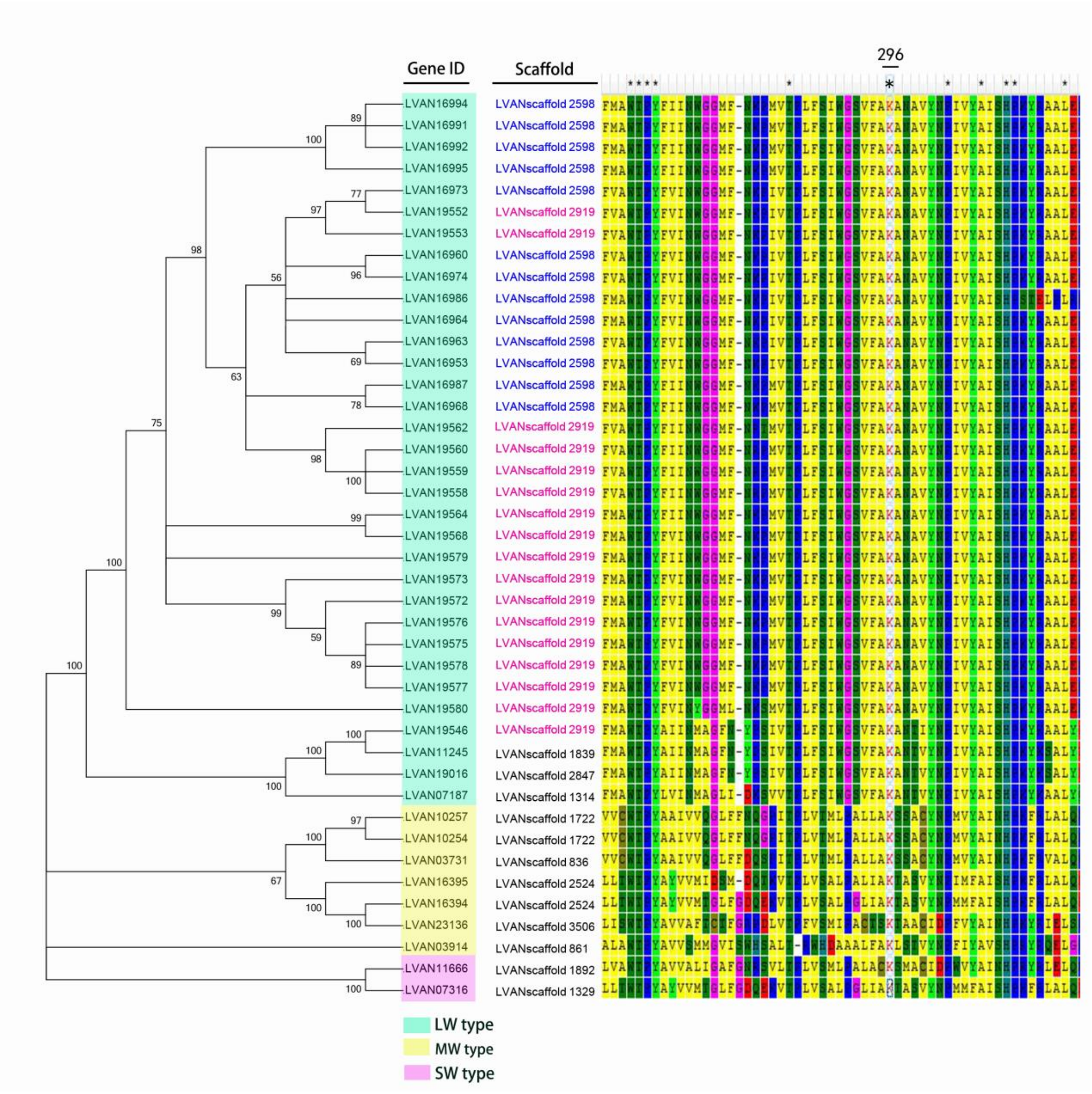

Supplementary Figure 18. Phylogenetic analysis and partial alignment of *L. vannamei* opsins.

Opsin peptides of *L. vannamei* cluster into three subfamilies in a neighbor-joining analysis with 1,000 bootstrap replicates. Branch support values are indicated next to crucial nodes. Gene ID is color-coded to indicate their subfamily corresponding to the long-wavelength type (LW), middle-wavelength type (MW) and short-wavelength type (SW). The scaffold of opsin genes is indicated in the middle panel, while the amino acid alignment is shown in the right panel. Conserved sites are indicated by asterisks on the top, while the key lysine residue (K296) necessary for the Schiff base formation is highlighted.

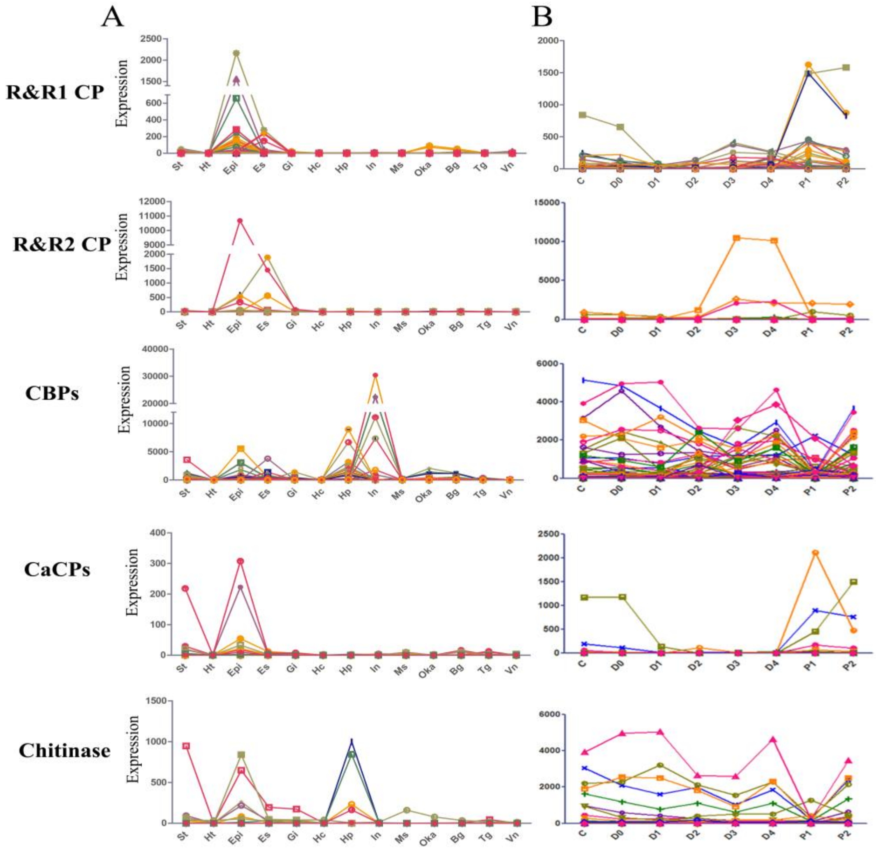

Supplementary Figure 19. The expression patterns of structural protein genes in different tissues (A) and molting stages (B).

Structural protein: cuticle proteins (including R&R1 type CP and R&R2 type CP), chitin binding proteins (CBPs), calcification related cuticular proteins (CaCPs). Tissues: St, stomach. Ht, heart. Epi, epidermis. Es, eyestalk. Gi, gill. Hc, hemocyte. Hp, hepatopancreas. In, intestine. Ms, muscle. Oka, lymphoid organ. Bg, brain ganglion. Tg, thoracic ganglion. Vn, ventral ganglion.

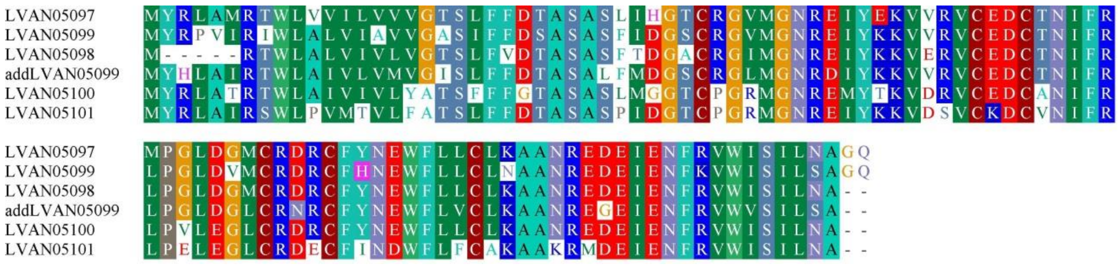

Supplementary Figure 20. Sequence alignment of MIH peptides in Scaffold1036.

Alignment of the full length (including signal peptide) amino acid sequence of six MIH genes in Scaffold1036 indicates six cysteine sites that are highly conserved in the CHH gene family.

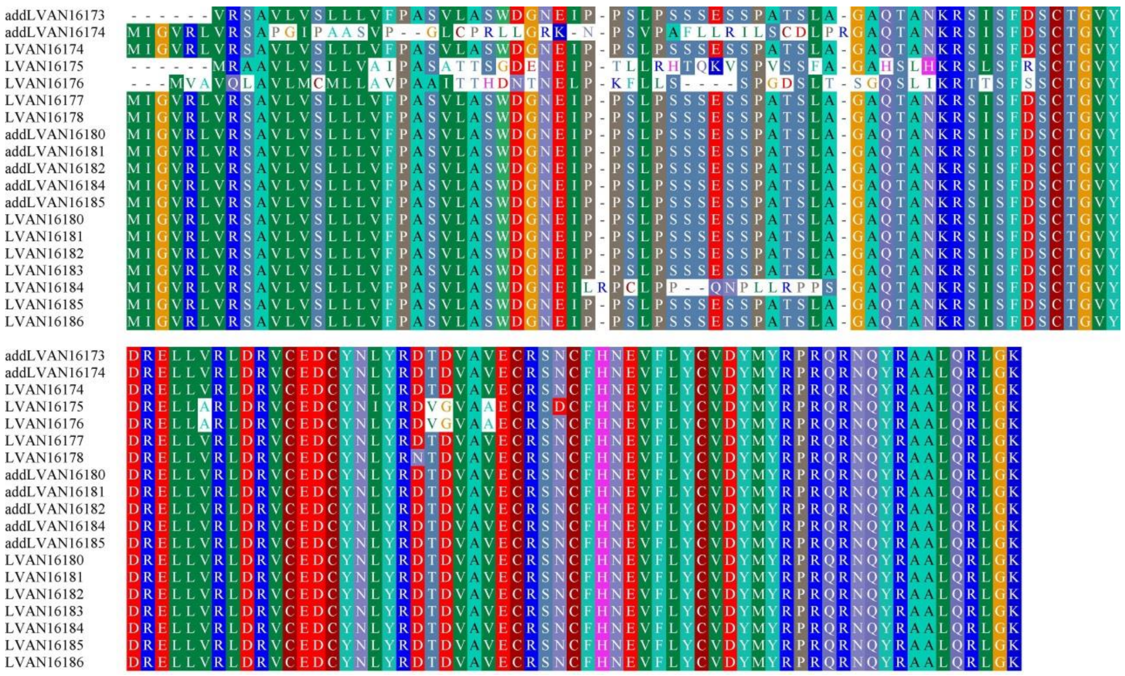

Supplementary Figure 21. Sequence alignment of type I CHH peptides in Scaffold2490.

Alignment of the full length (including signal peptide) amino acid sequence of CHH genes in Scaffold2490 indicates six cysteine sites that are highly conserved in the CHH gene family, and amindation at the C terminal which is associated with hyperglycemic activities.

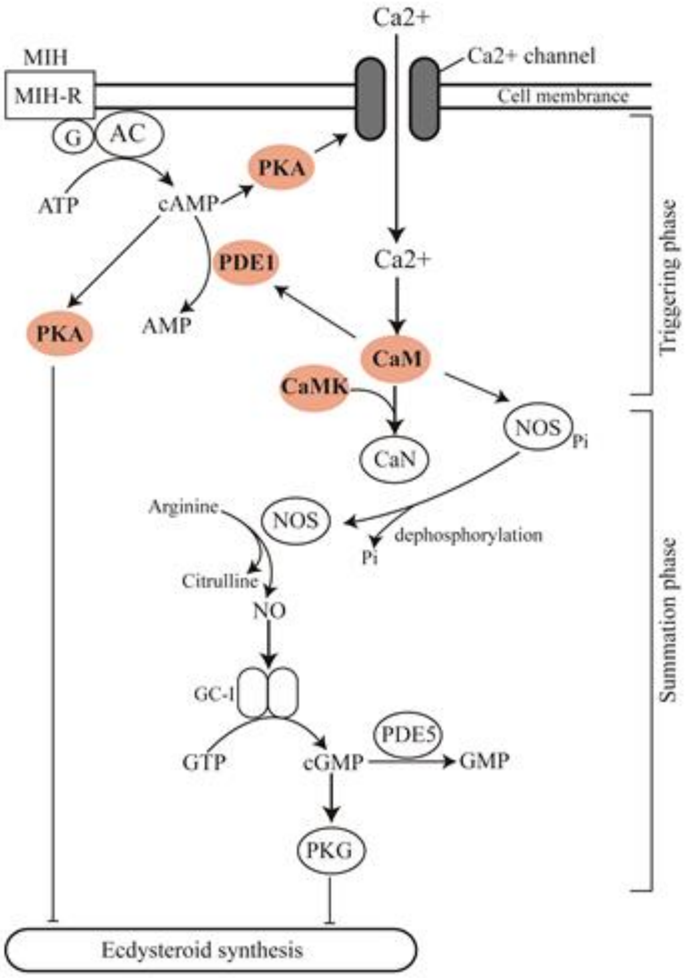

Supplementary Figure 22. Positively selected genes related to MIH signaling pathway in *L. vannamei*.

The circles with red backgrounds represent the positively selected genes in *L. vannamei*. Modified from Chang and Mykles[2], and Covi[3].

SREs	TCACNCCAC[1]	ACTACANNTCCC[2]
MIHS1036-3	TCAAGCCAC	ACCACACCCCCT
MIHS1036-4	GCATGCCAC	ACTATAAATACC
MIHS1036-6	TCGCAGCAC	ACTACA-TTCCC
MIHC1028	TCACAGCAC	ACCACACCACAC

Supplementary Figure 23. The putative sterol regulatory elements (SREs) located in the regulatory sequence of different MIH genes.

The conserved nucleotides of putative and reported SREs[4,5] are in red.

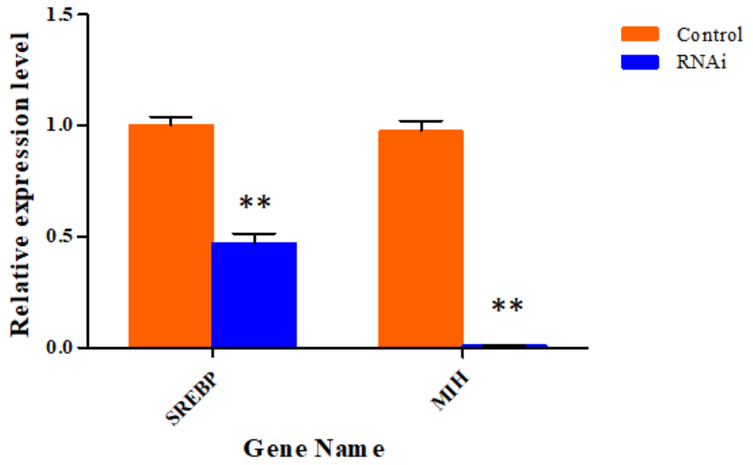

Supplementary Figure 24. DsRNA-mediated silencing efficiency of SREBP and MIH.

The expression of SREBP and MIH in eyestalk of *L. vannamei* was detected by qPCR with the primer pairs SREBP-qF/SREBP-qR and MIH1036-6qF/MIH1036-6qR listed in supplementary Table 31. Control: eyestalk samples from shrimp injected with dsEGFP. RNAi: eyestalk samples from shrimp injected with dsSREBP and dsMIH, respectively. Significant difference at P <0.01 of the expression level in two treatments was shown with two stars (**).Three replicates were performed in each treatment. Error bars standed the Standard Error of the Mean (SEM).

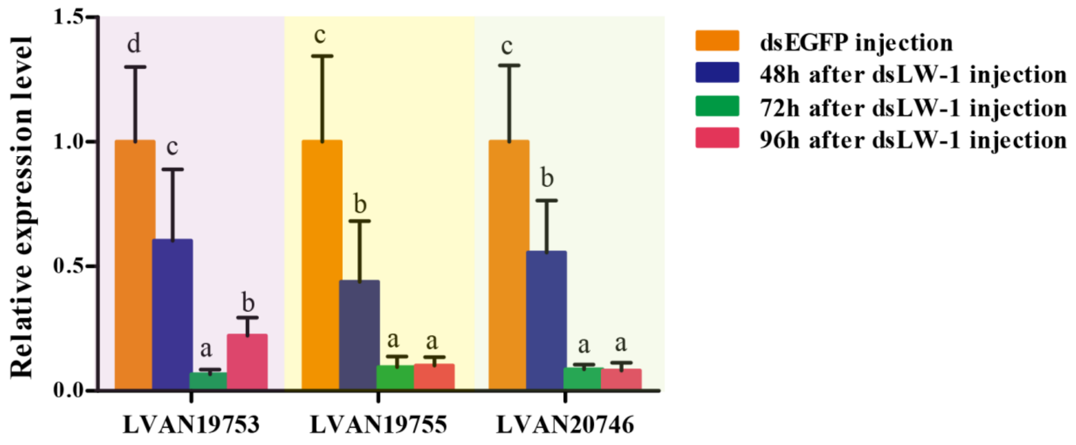

Supplementary Figure 25. dsRNA-mediated silencing of opsin genes over time after LW-1 opsin gene RNAi treatments.

Relative expressions of three opsin genes were detected at 48, 72 and 96 hours after knockdown of the LW-1 opsin gene (Supplementary Table 33). The expressions of three opsins after injection of dsEGFP were used as negative control treatments. Different lower-case letters indicate significant differences (P < 0.01) among different treatments. Three replicates were performed in each treatment. Error bars standed the Standard Error of the Mean (SEM).

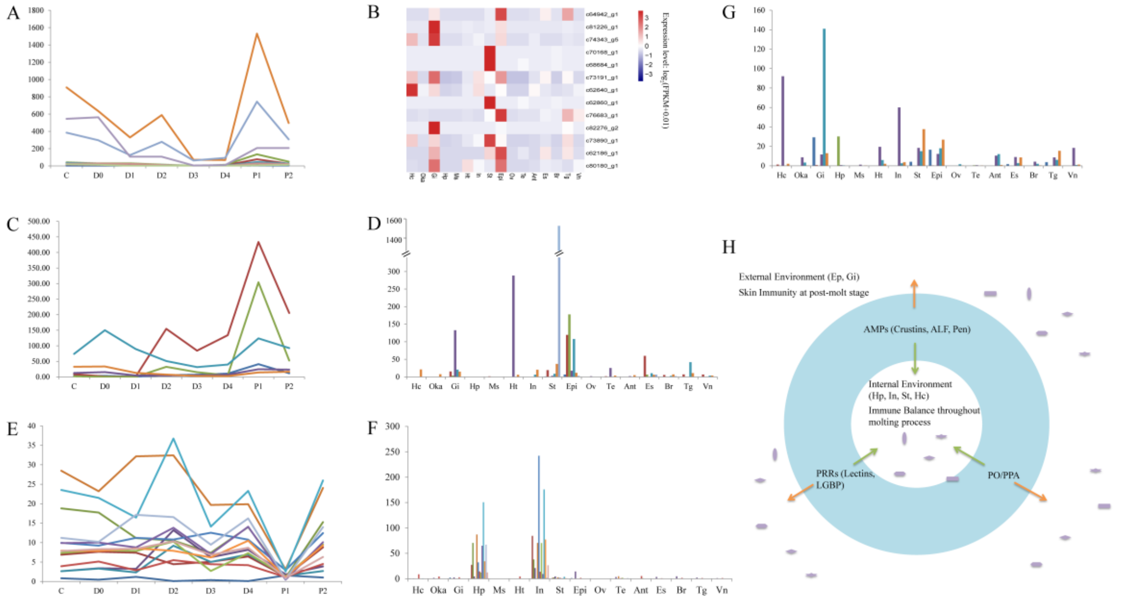

Supplementary Figure 26. Immune-related genes in the molting stages.

A. Expression patterns of crustin genes during molting stages. **B.** Tissue distribution of crustin genes which were differentially expressed during molting process. **C.** Expression patterns of selected lectin genes highly expressed at the post-molt stage. **D.** Tissue distribution of selected lectin genes highly expressed at the post-molt stage. **E.** Expression patterns of apoptosis related genes during molting stages. **F.** Tissue distribution of apoptosis related genes differentially expressed during molting process. **G.** Tissue distribution of phenoloxidase system genes differentially expressed during molting process. **H.** Immune protection of shrimp during molting process. C, intermolt. D0~D2, early pre-molt. D3 and D4, late pre-molt. P1 and P2, post-molt. Hc, hemocytes. Oka, lymphoid organ. Gi, gill. Hp, hepatopancreas. Ms, muscle. Ht, heart. In, intestine. St, stomach. Epi, epidermis. Ov, ovary. Te, testis. Ant, antennal gland. Es, eyestalk. Br, brain. Tg, thoracic ganglion. Vn, ventral ganglion.

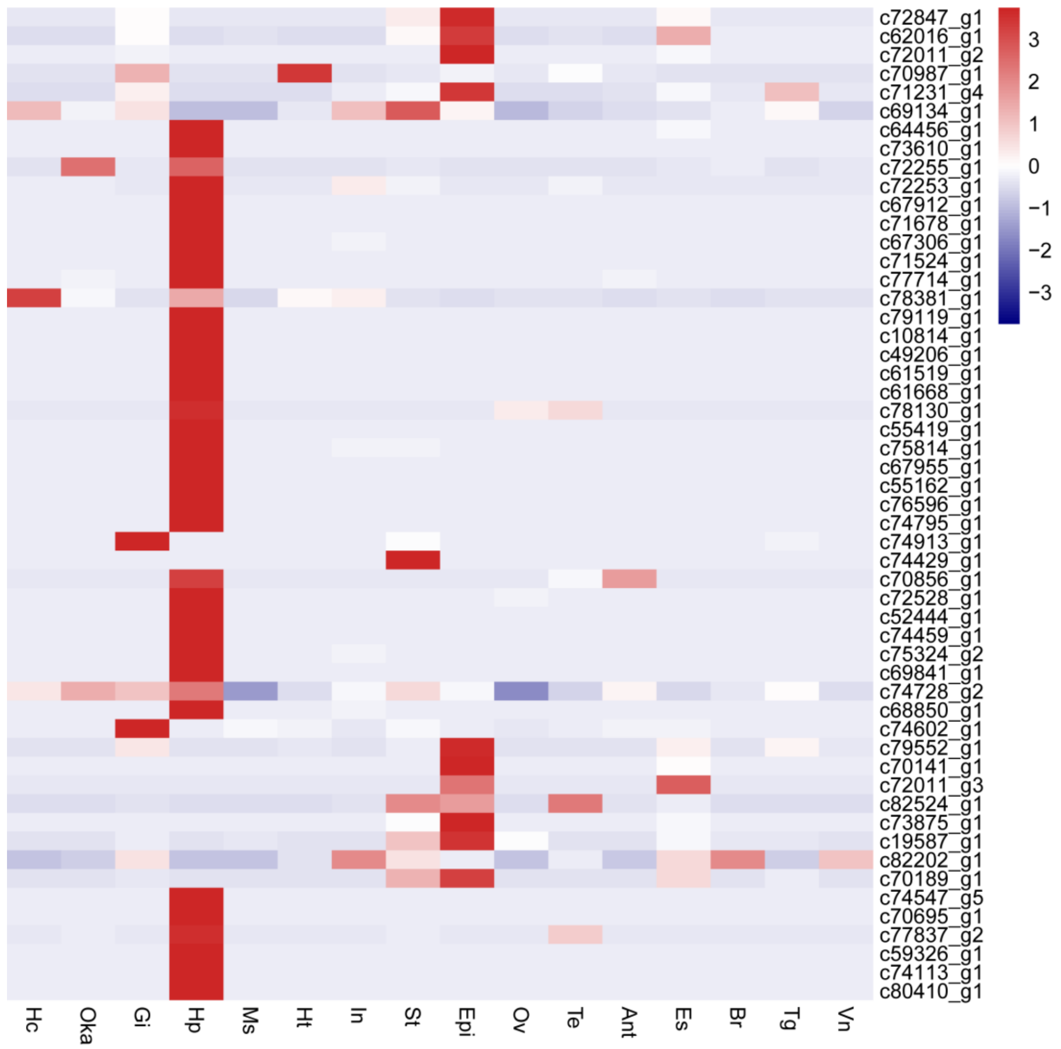

Supplementary Figure 27. Tissue distribution of lectin genes differentially expressed during the molting process.

Hc, hemocytes. Oka, lymphoid organ. Gi, gill. Hp, hepatopancreas. Ms, muscle. Ht, heart. In, intestine. St, stomach. Epi, epidermis. Ov, ovary. Te, testis. Ant, antennal gland. Es, eyestalk. Br, brain. Tg, thoracic ganglion. Vn, ventral ganglion.

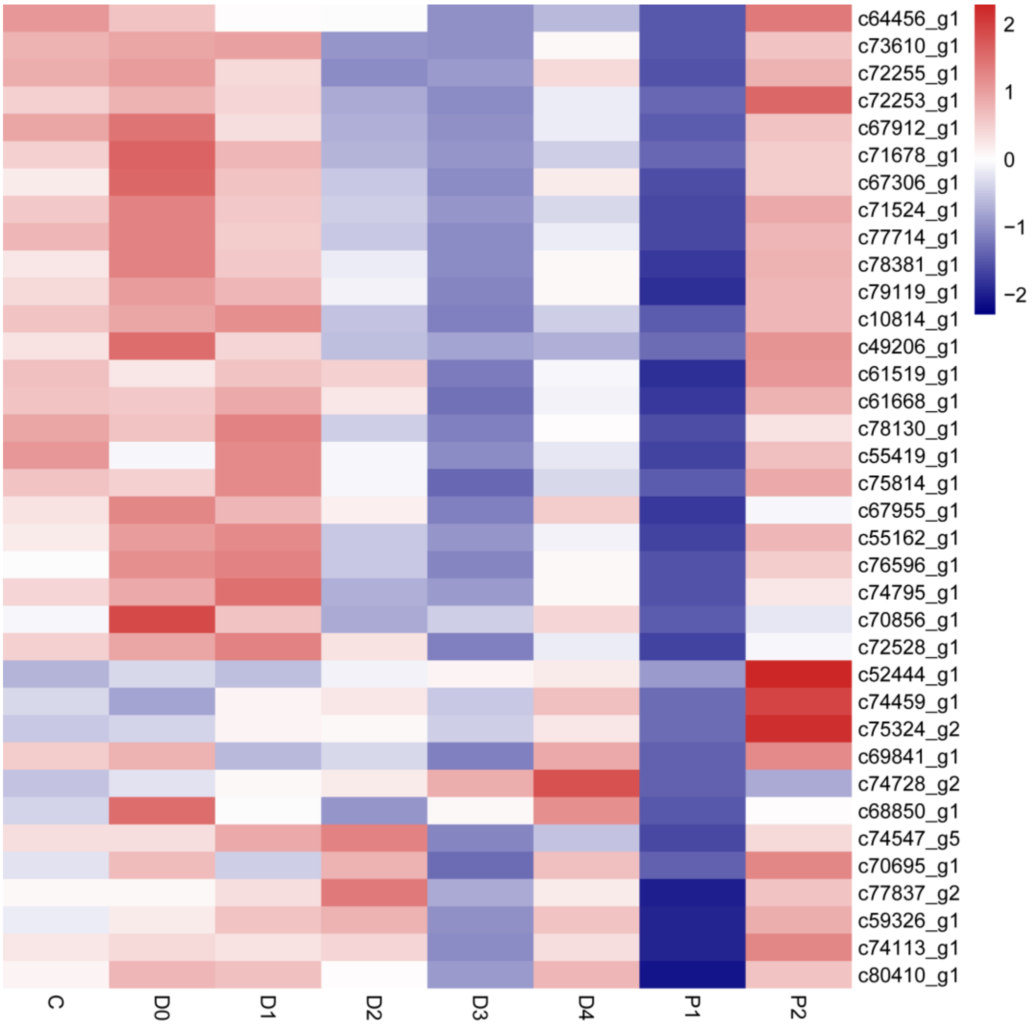

Supplementary Figure 28. Expression patterns of highly expressed lectin genes in hepatopancreas during the molting stages.

C, inter-molt. D0~D2, early pre-molt. D3 and D4, late pre-molt. P1 and P2, post-molt.

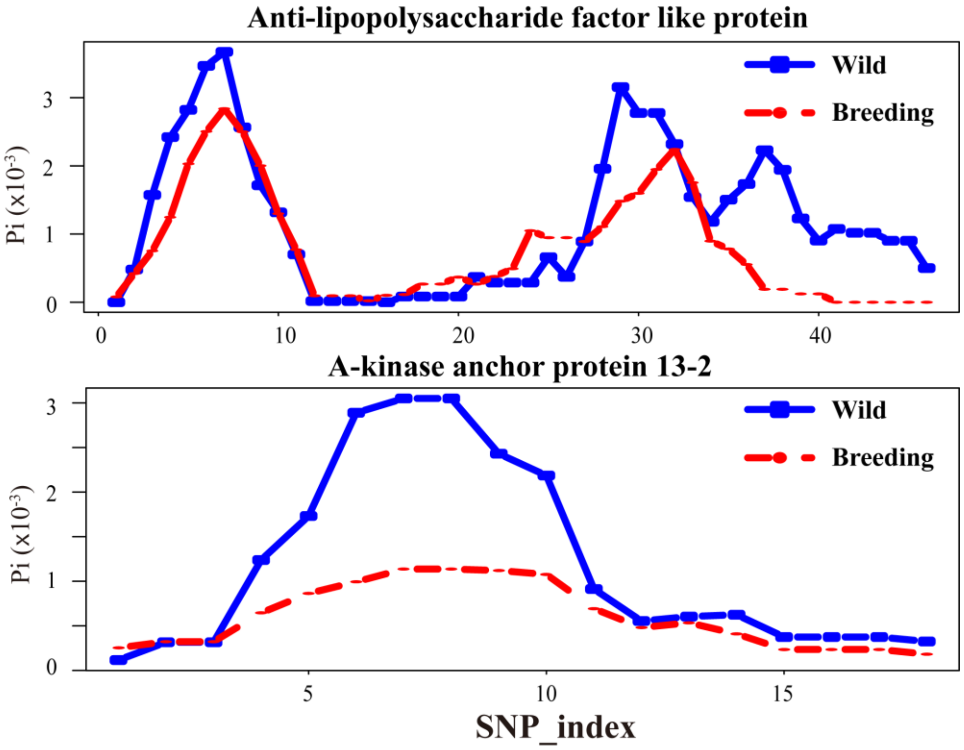

Supplementary Figure 29. The genetic diversity value θπ in wild and breeding population for the genes encoding anti-lipopolysaccharide factor like protein(ALF) andA-kinase anchor protein 13-2 (AKAP13-2).

A sliding-window approach (2 kb windows with 400 bp increments) was used to calculate θπ of every window for each gene, the SNP-index indicate the sliding-window of each gene.

Supplementary Tables

Supplementary Table 1. Statistics of Illumina sequencing data

Insert size (bp)	Reads Length （bp）	Total Raw Bases (Gb)	Total Clean bases (Gb)	Depth (×)*
180	100_100	254.89	213.09	86.98
300	100-100	159.32	148.48	60.60
500	100_100	251.05	227.12	92.70
800	100_100	57.15	47.91	19.56
1000	80_80	13.54	12.32	5.03
2000	100_100	83.52	75.42	30.78
5000	100_100	68.75	51.44	21.00
10000	100_100	43.30	37.40	15.27
20000	100_100	23.12	15.15	6.18
Total			**828.33**	**338.09**

* Genome size ~2.45 Gb

Supplementary Table 2. Statistics of PacBio sequencing data

Step	Mean length	Data	Coverage (×)*
Lvhn02	5,625	2,862,452,927	1.17
Lv1_1	6,551	25,490,552,307	10.40
Lv1_2	12,748	26,814,636,230	10.94
Lv1_3	12,432	25,286,562,106	10.32
Lv1_4	10,478	52,409,584,996	21.39
**Total**		**132,863,788,566**	**54.23**

* Genome size ~2.45 Gb

Supplementary Table 3. Statistics of BAC end sequencing data

Number of BAC clones sequenced	18828
Number of ends sequenced(pre-processed)	34,266 (28,993)
Paired-end sequences	11,972*2
Total length of sequences (bp)	11278277
Percentage of sequenced genome	0.0046
GC content	0.3948
Average length of sequences (bp)	389

Supplementary Table 4. Comparison of six assembly methods*

		SOAPdenovo	DBG2OLC	Falcon	SmartDenovo	HABOT	WTDBG
Contig	Number	811,715	43,938	463,151	60,355	110,906	50,304
	Total length	1,348,135,370	1,299,024,195	1,588,197,701	1,775,688,090	1,686,653,167	1,618,026,442
	Longest	1,219,275	707,549	1,219,275	421,901	214,418	739,419
	Shortest	100	1,349	300	1,062	100	63
	N50	2,826	43,564	9,496	34,826	25,477	57,650
	N90	712	13,276	1,271	15,383	9,552	14,641
	>2kb	195,846	43,927	165,107	60,301	90,055	47,569
Scaffold	Number	145,352		314,564		36,276	4,683
	Total length	1,895,785,128		1,804,586,902		1,902,472,206	1,663,559,157
	Longest	2,851,279		1,219,275		9,249,119	3,458,369
	Shortest	315		300		70	1,028
	N50	124,644		23,547		398,269	605,555
	N90	10,570		2,315		18,231	204,841
	>2kb	46,374		114,313		30,967	4,618

* The assembly results of SOAPdenovo were based on the Illumina sequencing data only. The assemblies of the other five different assembly methods, including DBG2OLC, Falcon, SmartDenovo, HABOT, WTDBG, were based on the PacBio data. As the contigs assembled by DBG2OLC and SmartDenovo showed poor accuracy and completeness, it was not used for the scaffolding.

Supplementary Table 5. Genome assembly comparison among five crustaceans.

Species	Genome size (Gb)	Scaffold N50 (Kb)	Longest scaffold (Mb)	Contig N50 (Kb)	Longest contig (Kb)
L. vannamei	2.6	605.555	3.458369	57.65	739.419
D. pulex	0.2	642.089	4.19303	47.461	528.83
P. hawaiensis	3	69.178	1.285385	4.003	141.947
P. virginalis	3.5	39.275	0.717999	0.745	69.465
E. sinensis	1.66	111.755	2.002076	2.666	100.563

The assembly of *L. vannamei* and *D. pulex* both showed high quality that the contig N50 and scaffold N50 as well as the longest contig and scaffold were all much longer than the other three crustaceans. However, *L. vannamei* has significantly larger genome size and more SSR contents than *D. pulex,* indicating it has a higher complex genome structure than *D. pulex, E. sinensis*[6].

Supplementary Table 6. Illumina reads coverage on assembly genome

Reads	Number of clean reads	391,866,685
	Percentage of mapped reads	93.25%
Genome	Coverage（%）	87%
	Coverage at least 5×（%）	76%
	Coverage at least 10×（%）	70%
	Coverage at least 20×（%）	62%
	Coverage at least 50×（%）	45%

Supplementary Table 7. Transcriptome unigene coverage on assembly genome.

Analysis	Unigenes
Unigene_number	71,703
Match_gene_number	67,725
Match_gene_percentage	94.45%
90%_in_one_scaf_number	44,853
90%_in_one_scaf_percentage	62.55%
50%_in_one_scaf_number	62,315
50%_in_one_scaf_percentage	86.91%

Supplementary Table 8. Completeness of the 248 core eukaryotic genes.

	#Prots	%Completeness	#Total	Average	%Ortho
Complete	191	77.02	241	1.26	21.99
Group 1	50	75.76	61	1.22	22.00
Group 2	43	76.79	48	1.12	11.63
Group 3	44	72.13	59	1.34	27.27
Group 4	54	83.08	73	1.35	25.93
Partial	235	94.76	350	1.49	36.60
Group 1	62	93.94	87	1.40	33.87
Group 2	53	94.64	71	1.34	28.30
Group 3	56	91.80	93	1.66	44.64
Group 4	64	98.46	99	1.55	39.06

Supplementary Table 9. Linkage map coverage on assembly genome.

Total markers	9,332
Quantity of scaffolds anchored on linkage groups	3,275
Total length of scaffolds anchored on linkage groups	1,449,959,403
Percentage of scaffolds anchored on linkage groups	87.34%

Supplementary Table 10. Summary of repetitive sequences.

Sequences:		4,683		
Total length:		1,663,565,344 bp (1,618,053,217 bp excluding N)		
Bases masked:		821,412,354 bp (49.38 %)		
		Number	Length	Percent
SINEs:		2,934	1,021,571	0.06%
LINEs:		126,578	46,932,128	2.82%
	RTE-BovB	22,215	12,816,134	0.77%
	Penelope	26,094	7,406,171	0.45%
LTR elements:		36,163	10,259,678	0.62%
	ERV_classI	4,291	353,176	0.02%
	Gypsy	8,747	3,659,760	0.22%
DNA elements:		932,683	155,145,405	9.33%
	hAT-Charlie	140,434	15,746,723	0.95%
	En-Spm	975,260	106,281,063	6.39%
	Maverick	52,513	13,342,433	0.80%
Unclassified:		216,901	55,650,866	3.35%
Total interspersed repeats:			269,009,648	16.17%
Satellites:		13,527	1,550,312	0.09%
Simple repeats:		5,515,074	398,243,493	23.93%
Low complexity:		1,103,699	154,532,775	9.29%

Supplementary Table 11. Simple sequence repeats in different species[7-17].

	Species	Common name	Percent (%)
Plants	*B. distachyon*	Purple false brome	1.89
	S. bicolor	Sorghum	2.49
	O. sativa	Asian rice	2.9
	Z. mays	Ma ź	0.86
	A. thaliana	Thale cress	0.45
	T. urartu	Einkorn wheat	1.21
Vertebrate	*F. peregrinus*	Peregrine	0.01
	F. cherrug	Saker	0
	T. guttata	Zebrafinch	0
	G. gallus	Chicken	0
	D. rerio	Zebrafish	0
	L. crocea	Large yellow croaker	1.72
	T. rubripes	Tiger puffer	1.74
	H. sapiens	Human	0.84
Insect	*L. migratoria*	Locust	0.2
	D. melanogaster	Fruit fly	1.08
	A. gambiae	Mosquitoes	1.08
	P. humanus	Human lice	10.52
	A. mellifera	Cape honey bee	1.92
Other Invertebrate	*C. elegans*	Roundworm	0.35
	H. robusta	Leech	6.36
	C. teleta	Polychaete	1.09
	C. gigas	Pacific oyster	0.72
	L. gigantea	Owl limpet	0.55
	L. vannamei	Pacific white shrimp	23.93
	D. pulex	Water flea	0.78
Fungi	*N. crassa*	Red bread mold	0.82
	S. cerevisiae	Yeast	0.36
	T. pseudonana	Marine centric diatom	0.23

Supplementary Table 12. Comparison of repetitive sequences among four crustaceans[14,18,19].

	L. vannamei	*P. virginalis*	*P. hawaiensis*	*D. pulex*
Total length:	1.66 Gb	3.29 Gb	4.02 Gb	197 Mb
Bases masked:	821 Mb	875 Mb	1.49 Gb	40 Mb
Repeat percent:	49.38%	26.59%	37.17%	20.45%
SINEs:	0.06%	0.80%	0.03%	0.98%
LINEs:	2.82%	9.52%	6.43%	0.90%
RTE-BovB	0.77%	0.12%	0.19%	0.24%
Penelope	0.45%	0.74%	1.02%	0.02%
L3/CR1	0.25%	5.80%	3.31%	0.00%
LTR elements:	0.62%	2.50%	0.58%	5.48%
ERV_classI	0.02%	0.00%	0.02%	0.00%
Gypsy	0.22%	0.79%	0.00%	2.77%
DIRS	0.00%	1.49%	0.03%	0.28%
DNA elements:	9.33%	0.62%	4.49%	1.75%
En-Spm	6.39%	0.01	0.05%	0.25%
TcMar-Tc1	0.03%	0.00%	1.93%	0.05%
Maverick	0.80%	0.07%	0.15%	0.00%
Charlie	0.95%	0.03	0.13%	0.00%
Tigger	0.04%	0.33%	0.05%	0.02%
Unclassified:	3.35%	11.88%	24.31%	10.22%
Total interspersed repeats:	16.17%	25.32%	35.84%	19.33%
Satellites:	0.09%	0.04%	0.04%	0.00%
Simple repeats:	23.93%	0.99%	1.27%	0.44%
Low complexity	9.29%	0.32%	0.13%	0.67%

Supplementary Table 13. Comparison of gene structures among three crustaceans[14,18,19]

Gene structure	*L. vannamei*	*P. hawaiensis*	*D. pulex*
Gene number	25,572	30,603	35,837
Gene average length	8,888.69	17,955.15	1,828.70
Gene Max length	329,769	623,480	114,502
Gene Min length	165	18	58
Gene length < 500bp	336	5,621	10,092
CDS average length	1,546.31	2,041.23	1,074.25
CDS Max length	35,259	28,449	25,464
CDS Min length	160	150	58
Exon number per gene	5.94	3.64	3.97
Exon average length	259.95	220.08	211.65
Intron average length	1,483.77	6,067.77	301.27

Supplementary Table 14. Noncoding small RNAs in the *L. vannamei* genome

Category	Name(copy number)
antisense	RNAI(1)
Cis-regulatory	Histone3(51), Hsp83_3_UTR(3), K_chan_RES(4), RRE(1)
Intron	Intron_gpII(1)
lncRNA	Sphinx_1(2), Sphinx_2(1)
ribozyme	RNase_MRP(2), RNaseP_arch(2), RNaseP_nuc(86)
rRNA	5_8S_rRNA(38), LSU_rRNA_archaea(44), LSU_rRNA_bacteria(48), LSU_rRNA_eukarya(75), PK-G12rRNA(25), SSU_rRNA_archaea(54), SSU_rRNA_bacteria(30), SSU_rRNA_eukarya(85), SSU_rRNA_microsporidia(65)
snoRNA	ACEA_U3(54), Fungi_U3(34), Plant_U3(51), SCARNA7(1), SCARNA8(2), snoMe28S-Cm2645(1), SNORA23(1), SNORA3(1), SNORA53(6), SNORA58(1), SNORA73(5), SNORA74(2), SNORA79(2), SNORA9(2), SNORD15(3), SNORD18(1), SNORD27(3), SNORD31(10), SNORD38(1), snosnR61(1), sR5(1), U3(56), SNORA23(1), SNORA3(1), SNORA53(6), SNORA58(1), SNORA73(5), SNORA74(2)
snRNA	ACEA_U3(54), Fungi_U3(34), Plant_U3(51), sR5(1), U12(1), U3(56), U4(9), U5(44), U6(35), U6atac(11)
tRNA	tRNA(1394), tRNA-Sec(64)
miRNA	miR-1(1), miR-2(4), miR-7(2), miR-9(5), miR-10(1), miR-12(1), miR-29(1), miR-33(2), miR-34(2), miR-71(1), miR-79(3), miR-92(1), miR-100(1), miR-124(1), miR-133(1), miR-153(1), miR-184(2), miR-190(1), miR-193(1), miR-210(1), miR-235(1), miR-252(2), miR-263(2), miR-275(1), miR-276(1), miR-277(1), miR-278(1), miR-279(1), miR-281(1), miR-282(1), miR-285(1), miR-306(2), miR-317(1), miR-745(2), miR-750(1), miR-981(1), miR-993(1), miR-995(1), miR-1000(1), miR-1175(1), miR-2001(1), miR-2765(1), miR-2788(1), miR-3791(1), miR-7478(1), bantam(1), miR-iab-41(1), let-7(1), n1(1), n2(1), n3(2), n4(2), n5(1), n6(1), n7(1), n8(2), n9(2), n10(1), n11(1), n12(2), n13(1), n14(1), n15(1), n16(1), n17(2), n18(1), n19(1), n20(1), n21(2), n22(1), n23(1), n24(1), n25(1), n26(2), n27(1), n28(1), n29(2), n30(1), n31(1), n32(1), n33(1), n34(1), n35(1), n36(1), n37(1), n38(1), n39(1), n40(1), n41(1), n42(1), n43(1), n44(2), n45(1), n46(1), n47(1), n48(1), n49(2), n50(1), n51(2), n52(1), n53(1), n54(1), n55(1), n56(1), n57(1), n58(1), n59(1), n60(1), n61(2), n62(2), n63(1), n64(1), n65(2), n66(2), n67(1), n68(1), n69(2), n70(1), n71(1), n72(1), n73(1), n74(1), n75(1), n76(3), n77(1), n78(1), n79(1), n80(1), n81(1), n82(1), n83(1), n84(1), n85(1), n86(1), n87(2), n88(1), n89(1), n90(1), n91(1), n92(1), n93(1), n94(2), n95(1), n96(1), n97(1), n98(2), n99(1), n100(1), n101(1), n102(1), n103(1), n104(1), n105(2), n106(1), n107(1), n108(1), n109(1), n110(1), n111(1), n112(1), n113(1), n114(1), n115(1), n116(1), n117(3), n118(2), n119(1), n120(1), n121(2), n122(1), n123(1)

Supplementary Table 15. GO enrichment analysis of the *L. vannamei* specific gene families.

GO ID	Description	GeneRatio (602)	BgRatio (3944)	*P*-value	FDR
GO:0016020	membrane	391 (64.95%)	2239 (56.77%)	6.00E-06	1.55E-03
GO:0007156	homophilic cell adhesion via plasma membrane adhesion molecules	18 (1.92%)	55 (0.73%)	6.80E-05	4.48E-02
GO:0098609	cell-cell adhesion	18 (1.92%)	57 (0.76%)	1.14E-04	4.48E-02
GO:0098742	cell-cell adhesion via plasma-membrane adhesion molecules	18 (1.92%)	57 (0.76%)	1.14E-04	4.48E-02

Supplementary Table 16. KEGG enrichment analysis of the *L. vannamei* specific gene families.

Pathway ID	Pathway	DEGs (1163)	All genes (6581)	*P*-value	Q-value
ko00052	Galactose metabolism	22 (1.89%)	54 (0.82%)	5.60E-05	8.96E-03
ko04973	Carbohydrate digestion and absorption	19 (1.63%)	44 (0.67%)	6.90E-05	8.96E-03
ko00512	Mucin type O-glycan biosynthesis	14 (1.2%)	28 (0.43%)	9.10E-05	8.96E-03
ko04970	Salivary secretion	30 (2.58%)	89 (1.35%)	1.82E-04	1.24E-02
ko04924	Renin secretion	26 (2.24%)	74 (1.12%)	2.29E-04	1.24E-02
ko05030	Cocaine addiction	16 (1.38%)	37 (0.56%)	2.53E-04	1.24E-02
ko00500	Starch and sucrose metabolism	27 (2.32%)	81 (1.23%)	4.63E-04	1.95E-02
ko04726	Serotonergic synapse	31 (2.67%)	101 (1.53%)	9.08E-04	3.35E-02
ko04728	Dopaminergic synapse	28 (2.41%)	89 (1.35%)	1.04E-03	3.39E-02
ko04918	Thyroid hormone synthesis	26 (2.24%)	83 (1.26%)	1.65E-03	4.88E-02

Supplementary Table 17. Number of neuronal development regulatory genes in *L. vannamei* and other arthropods.

	Lva	Api	Aga	Ame	Bmo	Dpu	Dme	Isc	Lmi	Pha	Phu	Sma	Tur	Tca	Zne
G-protein-coupled receptors (GPCRs)	457	100	63	136	71	50	163	56	51	60	59	60	65	114	52
transient receptor potential(TRP) channel	53	5	2	8	3	2	6	0	5	29	3	0	0	7	8
Ionotropic Glutamate Receptors (iGluRs)	166	26	14	44	16	25	33	9	16	39	14	28	14	51	15
glycine receptors (GlyRs)	154	6	4	31	3	3	21	7	4	12	2	29	6	27	3
Innexin	21	14	7	7	9	7	26	6	7	12	5	9	26	16	4
protocadherins	47	5	3	12	6	3	11	2	6	3	3	1	3	8	1
C2H2 zinc-finger proteins (C2H2 ZNFs)	414	18	13	35	25	14	33	23	16	22	13	19	37	28	15
crustacyanin	101	0	0	0	0	0	0	0	0	1	0	0	0	0	0

Lva, *Litopenaeus vannamei*. Api, *Acyrthosiphon pisum*. Aga, *Anopheles gambiae*. Ami, *Apis mellifera*. Bmo, *Bombyx mori*. Dpu, *Daphnia pulex*. Dme, *Drosophila melanogaster*. Isc, *Ixodes scapularis*. Lmi, *Locust migratoria*. Pha, *Parhyale hawaiensis*. Phu, *Pediculus humanus*. Sma, *Strigamia maritime*. Tur, *Tetranychus urticae*. Tca, *Tribolium castaneum*. Zne, *Zootermopsis nevadensis.*

Supplementary Table 18. Number of ionotropic glutamate receptors (iGluR) genes in Metazoans.

Species	Total	NMDA	AMPA	Kainate	Delta	Undetermined subtype
Homo sapiens	18	7	4	5	2	0
Ciona intestinalis	11	2	1	1	2	5
Branchiostoma floridae	10	0	3	3	0	4
Strongylocentrotus purpuratus	10	0	2	2	0	6
Saccoglossus kowalevskii	11	4	1	1	1	4
Drosophila melanogaster	47	14	8	10	0	15
Daphnia pulex	12	2	0	4	2	4
Parhyale hawaiensis	75	5	0	23	7	40
Litopenaeus vannamei	169	13	9	78	66	3
Caenorhabditis elegans	15	2	3	4	4	2
Aplysia californica	13	2	0	0	0	11
Lottia gigantea	12	2	0	0	0	10
Nematostella vectensis	10	1	0	0	0	9
Trichoplax adhaerens	12	0	0	0	0	12
Amphimedon queenslandica	0	0	0	0	0	0
*Pleurobrachia bachei**	14	0	0	0	0	14
Arabidopsis thaliana	20	0	0	0	0	0

Supplementary Table 19. The statistics of structural proteins in *L. vannamei* and other 13 arthropods.

Category	Conserved domains		Lvan	Dpul	Apis	Agam	Amel	Bmor	Isca	Lmig	Phum	Smar	Turt	Tcas	Znev	Phaw
Cuticle proteins	Chitin_binding 4	R&R1 type	**69**	64	15	48	23	63	0	34	13	13	1	40	12	34
		R&R2 type	39	175	94	105	25	108	54	51	21	13	29	53	37	49
Chitin binding proteins	Chitin_binding 2		**190**	108	38	91	82	59	47	66	37	59	52	95	47	115
Chitinase			**18**	11	5	8	7	4	5	5	5	4	9	6	6	6
Calcified-related cuticle protein			**24**	0	0	0	0	0	0	0	0	0	0	0	0	1

Bolded text denotes significant gene expansion in *L. vannamei*.

The structural proteins were search with conserved domains by hmmscan (https://www.ebi.ac.uk/Tools/hmmer/search/hmmscan). Rebers and Riddiford (R&R)1 and R&R2 type cuticle proteins were distinguished by cuticleDB (http://bioinformatics2.biol.uoa.gr/cuticleDB/)

Species: Lvan, *L. vannamei*. Dpul, *Daphnia pulex*. Apis, *Acyrthosiphon pisum*. Agam, *Anopheles gambiae*. Amel,*Apis mellifera*. Bmor, *Bombyx mori*. Isca, *Ixodes scapularis*. Lmig, *Locust migratoria*. Phum, *Pediculus humanus*.Smar, *Strigamia maritime*. Turt, *Tetranychus urticae*. Tcas, *Tribolium castaneum*. Znev, *Zootermopsis nevadensis*. Phaw, *Parhyale hawaiensis*.

Supplementary Table 20. The broodstocks for resequencing.

Individual ID	Population origin	Population type
SIS1	Shrimp Improvement System	breeding line
SIS2	Shrimp Improvement System	breeding line
E1	Ecuador	breeding line
E2	Ecuador	breeding line
E3	Ecuador	breeding line
E4	Ecuador	breeding line
E5	Ecuador	breeding line
E6	Ecuador	breeding line
G1	Guihai No.1	breeding line
G2	Guihai No.1	breeding line
K1	Kehai No.1	breeding line
K2	Kehai No.1	breeding line
M1	Mexico	wild
M2	Mexico	wild
M3	Mexico	wild
M4	Mexico	wild
M5	Mexico	wild
M6	Mexico	wild
M7	Mexico	wild
M8	Mexico	wild
Z1	Charoen Pokphand	breeding line
Z2	Charoen Pokphand	breeding line

Supplementary Table 21. Statistics of clean reads of the resequencing genomes.

ID	Reads	Bases (bp)	Genome coverage (**x**)
E1	475,613,552	67,404,279,865	27.51
E2	506,334,312	71,739,501,543	29.28
E3	508,362,324	71,931,326,909	29.36
E4	537,178,858	80,249,639,077	32.75
E5	489,161,684	73,137,316,758	29.85
E6	566,680,220	79,412,500,937	32.41
G1	329,863,064	45,346,949,307	18.51
G2	341,464,730	46,952,009,382	19.16
K1	626,567,766	53,856,715,486	21.98
K2	341,464,730	47,101,703,732	19.23
M1	338,984,324	46,705,434,078	19.06
M2	330,505,384	45,637,285,901	18.63
M3	463,184,192	64,476,512,350	26.32
M4	348,462,830	48,458,337,730	19.78
M5	336,637,708	47,726,698,217	19.48
M6	318,726,982	43,267,340,603	17.66
M7	361,138,288	50,219,910,121	20.50
M8	398,668,036	55,060,030,921	22.47
S1	478,358,152	67,634,718,375	27.61
S2	516,645,214	72,818,933,335	29.72
Z1	328,323,444	44,574,314,624	18.19
Z2	463,621,742	65,446,427,009	26.71
Average	427,543,070	58,598,085,739	23.92

Supplementary Table 22. Statistics of variants identified in each pseudo-chromosome.

Chromosome	Length	Variants	Variants rate
LG1	52,152,945	1,026,941	50
LG2	16,719,668	321,544	51
LG3	66,465,400	1,297,714	51
LG4	18,181,660	395,375	45
LG5	13,261,821	275,530	48
LG6	22,619,616	485,080	46
LG7	16,413,103	311,149	52
LG8	19,842,451	387,536	51
LG9	43,829,617	799,522	54
LG10	32,884,954	655,600	50
LG11	24,369,793	491,093	49
LG12	18,265,359	372,264	49
LG13	36,838,812	732,378	50
LG14	3,362,702	67,335	49
LG15	49,763,323	1,022,646	48
LG16	46,225,372	943,912	48
LG17	48,762,776	915,275	53
LG18	16,029,459	312,773	51
LG19	27,576,248	559,985	49
LG20	35,335,268	699,153	50
LG21	3,572,971	76,099	46
LG22	15,721,327	305,037	51
LG23	8,001,244	145,170	55
LG24	32,142,749	603,220	53
LG25	48,751,593	954,580	51
LG26	30,086,496	597,053	50
LG27	39,677,014	732,142	54
LG28	43,658,239	878,821	49
LG29	44,455,973	861,651	51
LG30	21,955,514	431,115	50
LG31	44,805,893	889,836	50
LG32	60,100,041	1,190,897	50
LG33	21,849,204	438,615	49
LG34	37,011,837	719,088	51
LG35	16,919,992	337,830	50
LG36	31,429,309	610,481	51
LG37	34,088,060	662,377	51
LG38	31,085,751	579,343	53
LG39	16,351,714	344,893	47
LG40	43,147,990	851,049	50
LG41	30,076,848	611,869	49

LG42	71,142,342	1,395,022	50
LG43	56,310,883	1,132,981	49
LG44	38,023,466	780,722	48
UNCHR	235,123,190	1,026,941	50

Supplementary Table 23. Statistics of calling variants according variant type for population.

Type	Count	Percent
Intergenic	28,374,241	86.678%
Intron	3,844,540	11.744%
Splice site acceptor	1,400	0.004%
Splice site donor	1,452	0.004%
Splice site region	37,733	0.115%
Exon	475,948	1.454%

	Type	Count
Exon	synonymous_variant	261,056
	nonsynonymous_variant	206,026

Supplementary Table 24. Annotation of the genes subjected to the artificial selection pressure.

Gene name	Annotation
LVAN05453	anti-lipopolysaccharide factor like protein
LVAN05610	AGAP002111-PA
LVAN05608	NA
LVAN05611	synaptojanin-1, putative
LVAN05607	hypothetical protein DAPPUDRAFT_230283
LVAN05609	Centromere protein L, partial
LVAN05606	hypothetical protein DAPPUDRAFT_309342
LVAN09979	PREDICTED: tetratricopeptide repeat protein 31-like isoform X2
LVAN09980	hypothetical protein L798_04895, partial
LVAN10333	hypothetical protein DAPPUDRAFT_243907
LVAN10335	Follistatin-related protein 5
LVAN10332	NA
LVAN10334	hypothetical protein DAPPUDRAFT_243907
LVAN10541	PREDICTED: uncharacterized protein LOC105196560
LVAN10542	PREDICTED: la-related protein 6-like isoform X2
LVAN10540	PREDICTED: ubiquitin-conjugating enzyme E2 T
LVAN11583	PREDICTED: protocadherin-like wing polarity protein stan isoform X1
LVAN12735	hypothetical protein DAPPUDRAFT_240350
LVAN12737	A-kinase anchor protein 13
LVAN12736	A-kinase anchor protein 13
LVAN16762	NA
LVAN16764	hypothetical protein
LVAN16763	GH24651
LVAN19641	lactosylceramide 4-alpha-galactosyltransferase
LVAN19639	lactosylceramide 4-alpha-galactosyltransferase
LVAN19640	NA
LVAN19643	trafficking protein particle complex 3
LVAN19642	NA

Supplementary Table 25. Primer sequences and corresponding annealing temperature.

Primer name	Primer sequence（5'-3'）	Annealing temperature (℃)
MIH1036-3qF	GCATTCCTGCCGTCTCAAGC	56.4
MIH1036-3qR	CGATCTCGTCCTCCCTGTTG	
MIH1036-4qF	GCACTACATTCCCACCGTCTT	56.4
MIH1036-4qR	TCGCAGCCTTCAGACAGACC	
MIH1036-6qF	ACACCTTGCCTTTACTTCGT	56.4
MIH1036-6qR	TGGGTTCTTCTGGGCTCACT	
SREBP-qF	GCTTCTGGCGACACCGTAAA	56.4
SREBP-qR	TGGAGGATCTGCCGAGTTATC	
18S-qF	TATACGCTAGTGGAGCTGGAA	55
18S-qR	GGGGAGGTAGTGACGAAAAAT	
dsMIH1036-6F	TAATACGACTCACTATAGGGACACCTTGCCTTTACTTCGT	60
dsMIH1036-6R	TAATACGACTCACTATAGGGTGGGTTCTTCTGGGCTCACT	
dsEGFP-F	TAATACGACTCACTATAGGGCAGTGCTTCAGCCGCTACCC	60
dsEGFP-R	TAATACGACTCACTATAGGGAGTTCACCTTGATGCCGTTCTT	
Srebp-dsF	TAATACGACTCACTATAGGGGAGTCAAGCCTATGATTGTGCC	62
Srebp-dsR	TAATACGACTCACTATAGGGTCAGCCTGTTTACGGTGTCG	

Supplementary Table 26. The primer sequences used in the dsRNA-opsin silencing.

**Gene ID**	**Primer sequence**
LW-F+T7-1	TAATACGACTCACTATAGGTGCCTTCCACCATTCTTT
LW-R+T7-1	TAATACGACTCACTATAGGCCACCGTCTCTACCCTCA
LW-F-F+T7-2	TAATACGACTCACTATAGGCCTGCTAATCTTCTGGTC
LW-R+T7-2	TAATACGACTCACTATAGGTGTTCTCGCATTCCCTTC
LVAN19753 _F	CGTCTACAACCCCATCGT
LVAN19753 _R	CCACCGTCTCTACCCTCAG
LVAN19755 _F	CCCTTGCTTGGTGCCTTC
LVAN19755 _R	TTGTTCTCGCATTCCCTTCT
LVAN20746 _F	AAGCGAGGAAGCCCAGAA
LVAN20746 _R	CCCAGTTGATGACGAAGTAGG

Supplementary Reference:

1. Chow, S., Dougherty, W.J. & Sandifer, P.A. Meiotic Chromosome Complements and Nuclear-DNA Contents of 4 Species of Shrimps of the Genus Penaeus. *Journal of Crustacean Biology* 10, 29-36 (1990).
2. Chang, E.S. & Mykles, D.L. Regulation of crustacean molting: A review and our perspectives. *General and Comparative Endocrinology* 172, 323-330 (2011).
3. Covi, J.A., Chang, E.S. & Mykles, D.L. Conserved role of cyclic nucleotides in the regulation of ecdysteroidogenesis by the crustacean molting gland. *Comparative Biochemistry and Physiology a-Molecular & Integrative Physiology* 152, 470-477 (2009).
4. Seo, Y.K. *et al.* Genome-wide analysis of SREBP-1 binding in mouse liver chromatin reveals a preference for promoter proximal binding to a new motif. *Proceedings of the National Academy of Sciences of the United States of America* 106, 13765-13769 (2009).
5. Osborne, T.F. Sterol regulatory element-binding proteins (SREBPs): Key regulators of nutritional homeostasis and insulin action. *Journal of Biological Chemistry* 275, 32379-32382 (2000).
6. Song, L. *et al.* Draft genome of the Chinese mitten crab, Eriocheir sinensis. *Gigascience* 5, 5 (2016).
7. Zhan, X. *et al.* Peregrine and saker falcon genome sequences provide insights into evolution of a predatory lifestyle. *Nature Genetics* 45, 563-6 (2013).
8. Jia, J. *et al.* Aegilops tauschii draft genome sequence reveals a gene repertoire for wheat adaptation. *Nature* 496, 91-5 (2013).
9. Ling, H.Q. *et al.* Draft genome of the wheat A-genome progenitor Triticum urartu. *Nature* 496, 87-90 (2013).
10. Ao, J.Q. *et al.* Genome Sequencing of the Perciform Fish Larimichthys crocea Provides Insights into Molecular and Genetic Mechanisms of Stress Adaptation. *Plos Genetics* 11(2015).
11. Howe, K. *et al.* The zebrafish reference genome sequence and its relationship to the human genome. *Nature* 496, 498-503 (2013).
12. Wang, X.H. *et al.* The locust genome provides insight into swarm formation and long-distance flight. *Nature Communications* 5, 1-9 (2014).
13. Simakov, O. *et al.* Insights into bilaterian evolution from three spiralian genomes. *Nature* 493, 526-531 (2013).
14. Colbourne, J.K. *et al.* The ecoresponsive genome of Daphnia pulex. *Science* 331, 555-61 (2011).
15. Zhang, G. *et al.* The oyster genome reveals stress adaptation and complexity of shell formation. *Nature* 490, 49-54 (2012).
16. Armbrust, E.V. *et al.* The genome of the diatom Thalassiosira pseudonana: ecology, evolution, and metabolism. *Science* 306, 79-86 (2004).
17. Jiang, Q., Li, Q., Yu, H. & Kong, L.F. Genome-Wide Analysis of Simple Sequence Repeats in Marine Animals-a Comparative Approach. *Marine Biotechnology* 16, 604-619 (2014).
18. Gutekunst, J. *et al.* Clonal genome evolution and rapid invasive spread of the marbled crayfish. *Nature Ecology & Evolution* 2, 567-573 (2018).
19. Kao, D. *et al.* The genome of the crustacean Parhyale hawaiensis, a model for animal development, regeneration, immunity and lignocellulose digestion. *elife* 5(2016).
