# Supplementary material for: Penaeid shrimp genome provides insights into benthic adaptation and frequent molting
Source: Nat Commun. 2019 Jan 21;10:356. doi: 10.1038/s41467-018-08197-4 (PMC6341167; doi:10.1038/s41467-018-08197-4)
Supplement: Supplementary file 3 — Description of Additional Supplementary Files [file 41467_2018_8197_MOESM3_ESM.pdf]

Supplementary Data 1.
KEGG enrichment analysis of the L. vannamei expanded gene families.

Supplementary Data 2.
GO enrichment analysis of the L. vannamei expanded gene families

Supplementary Data 3.
Apolipoprotein family genes expressed in different tissues.

Supplementary Data 4.
Expression patterns (FPKM) of crustacean hyperglycemic hormone (CHH) genes transcripts in different tissues and molting stages of L. vannamei.

Supplementary Data 5. C
rustacean hyperglycemic hormone (CHH) genes in the genome of L. vannamei.

Supplementary Data 6.
The positively selected genes in the L. vannamei genome.

Supplementary Data 7.
Expression patterns (RPKM in the transcriptome) of differentially expressed immune-related genes at different molting stages of L. vannamei.

Supplementary Data 8.
The source data underlying figures in the manuscript.
